# A new design for a green calcium indicator with a smaller size and a reduced number of calcium-binding sites

**DOI:** 10.1038/srep34447

**Published:** 2016-09-28

**Authors:** Natalia V. Barykina, Oksana M. Subach, Danila A. Doronin, Vladimir P. Sotskov, Marina A. Roshchina, Tatiana A. Kunitsyna, Aleksey Y. Malyshev, Ivan V. Smirnov, Asya M. Azieva, Ilya S. Sokolov, Kiryl D. Piatkevich, Mikhail S. Burtsev, Anna M. Varizhuk, Galina E. Pozmogova, Konstantin V. Anokhin, Fedor V. Subach, Grigori N. Enikolopov

**Affiliations:** 1 NBICS Department, Moscow Institute of Physics and Technology, Moscow 123182, Russia; 2 Department of Systems Neurobiology and Functional Neurochemistry, P.K. Anokhin Institute of Normal Physiology of RAMS, Moscow 125009, Russia; 3 National Research Center “Kurchatov Institute”, Moscow 123182, Russia; 4 Laboratory of Cellular Neurobiology of Learning, Institute of Higher Nervous Activity and Neurophysiology of RAS, Moscow 117485, Russia; 5 Medico-Biological Faculty, N.I. Pirogov Russian National Research Medical University, Moscow 117997, Russia; 6 MIT Media Lab, Massachusetts Institute of Technology, Cambridge, MA 02139, USA; 7 Federal Research and Clinical Center of Physical-Chemical Medicine of Federal Medical Biological Agency, Moscow 119435, Russia; 8 Engelhardt Institute of Molecular Biology RAS, Moscow 119991, Russia; 9 Cold Spring Harbor Laboratory, Cold Spring Harbor, NY 11724, USA; 10 Department of Anesthesiology, Stony Brook University Medical Center, NY 11794, USA; 11 Center for Developmental Genetics, Stony Brook University, NY 11794, USA

## Abstract

Genetically encoded calcium indicators (GECIs) are mainly represented by two- or one-fluorophore-based sensors. One type of two-fluorophore-based sensor, carrying *Opsanus* troponin C (TnC) as the Ca^2+^-binding moiety, has two binding sites for calcium ions, providing a linear response to calcium ions. One-fluorophore-based sensors have four Ca^2+^-binding sites but are better suited for *in vivo* experiments. Herein, we describe a novel design for a one-fluorophore-based GECI with two Ca^2+^-binding sites. The engineered sensor, called NTnC, uses TnC as the Ca^2+^-binding moiety, inserted in the mNeonGreen fluorescent protein. Monomeric NTnC has higher brightness and pH-stability *in vitro* compared with the standard GECI GCaMP6s. In addition, NTnC shows an inverted fluorescence response to Ca^2+^. Using NTnC, we have visualized Ca^2+^ dynamics during spontaneous activity of neuronal cultures as confirmed by control NTnC and its mutant, in which the affinity to Ca^2+^ is eliminated. Using whole-cell patch clamp, we have demonstrated that NTnC dynamics in neurons are similar to those of GCaMP6s and allow robust detection of single action potentials. Finally, we have used NTnC to visualize Ca^2+^ neuronal activity *in vivo* in the V1 cortical area in awake and freely moving mice using two-photon microscopy or an nVista miniaturized microscope.

Optical techniques using genetically encoded calcium indicators (GECIs) based on fluorescent proteins (FPs) are broadly applied for *in vivo* visualization of neuronal activity. FP-based calcium indicators (or sensors) can be classified into two major designs (Fig. 1a).

The first class of GECIs includes the FRET (fluorescence resonance energy transfer)-based family of sensors, which is composed of two fluorescent proteins, one acting as a donor and another as an acceptor, with a Ca^2+^-binding domain located between them^1^. The latter can be represented by calmodulin (CaM) in combination with the M13 peptide from myosin light chain kinase (CaM/M13) or by a minimal Ca^2+^-binding motif from the C-terminal domain of troponin C (TnC). In the first type of FRET sensor, CaM carries four calcium ion-binding sites, and while this feature ensures the efficiency of CaM/M13-based GECIs, such sensors, acting as Ca^2+^ buffers, may affect the cellular concentration of free calcium ions^2^. Therefore, GECIs that would sequester fewer calcium ions are preferable. Indeed, an important advantage of the highly efficient Twitch family, a second type of FRET sensor, is that they have two calcium ion-binding sites per molecule, due to the use of TnC from the toadfish, *Opsanus tau*^3^. The reduced number of Ca^2+^-binding sites in GECIs is thought to provide a more linear response, thus facilitating quantification of changes in Ca^2+^ concentration^2^. We cannot exclude the possibility that it is not the reduced stoichiometry itself that provides a linear response of TnC but instead its structural properties, as reflected in the interaction between the two Ca^2+^-binding sites. While FRET-based GECIs are sensitive and specific, their use is potentially limited, in particular because of their large size.

The second type of GECI design is typically based on a single circularly permutated FP (cpFP) and CaM/M13-like peptides located in close proximity to the chromophore (Fig. 1a)^4^. Despite the popularity of the circularly permutated calcium indicators (e.g., of the GCaMP family), they have several limitations. For example, their CaM/M13 sensory components bind twice as many calcium ions as the Twitch family of FRET-based indicators (i.e., four calcium ions per molecule). Furthermore, because of low fluorescence at resting Ca^2+^ levels in neurons, most of these sensors require co-transfection with bright, spectrally distinct markers^5^. In addition, the pH sensitivity of cpFP-based GECIs may hinder unambiguous registration of Ca^2+^ dynamics because of the pH changes that can accompany neuronal activity^6^. Moreover, Ca^2+^ imaging using pH-sensitive GECIs is not perfectly compatible with all-optical neurophysiology experiments that involve depolarization of neurons with channelrhodopsins (ChRs) because of photo-induced acidification as a result of proton release by ChRs^7^.

Because the vast majority of current experiments with GECIs employ adeno-associated virus (AAV)-based vectors, whose packaging capacity is limited^8^, the size of the recombinant indicator is yet another common problem. While FRET calcium sensors have been optimized for reduction of Ca^2+^-binding sites, their design is still restricted to ~556 amino acids^3^. The cpFP-based sensors are smaller but still reach ~420 amino acids. Therefore, a smaller size would facilitate AAV packaging, particularly if additional domains must be added to the sensors.

To overcome the limitations of the current GECIs, we decided to engineer a sensor with a novel hybrid design that would combine the advantages of the sensory domain from the Twitch FRET sensors with the fluorescent domain of the cpFP-based sensors. In particular, the construct would consist of a single fluorescent protein and a minimal TnC motif as the sensor to reduce the size and the number of Ca^2+^-binding sites. Furthermore, such an improved sensor would be pH-stable and would have an increased baseline intensity, which would facilitate the choice of cells for recording. Herein, we present a calcium sensor with a novel design that combines the brightness and pH stability of mNeonGreen FP with the small size and reduced number of Ca^2+^-binding sites of the TnC motif. This sensor, referred to as NTnC, has high baseline intensity and an inverted fluorescence response, thus facilitating the choice of neurons for recording Ca^2+^ dynamics. We validate the NTnC indicator *in vitro*, in mammalian cells, and in neuronal cultures using confocal microscopy, multi-electrode arrays (MEAs), and whole-cell patch clamp recordings and in awake mice *in vivo* using both two-photon microscopy and an nVista miniaturized microscope.

## Results and Discussion

### Development of a novel green fluorescent calcium sensor, NTnC

**Figure 1:**
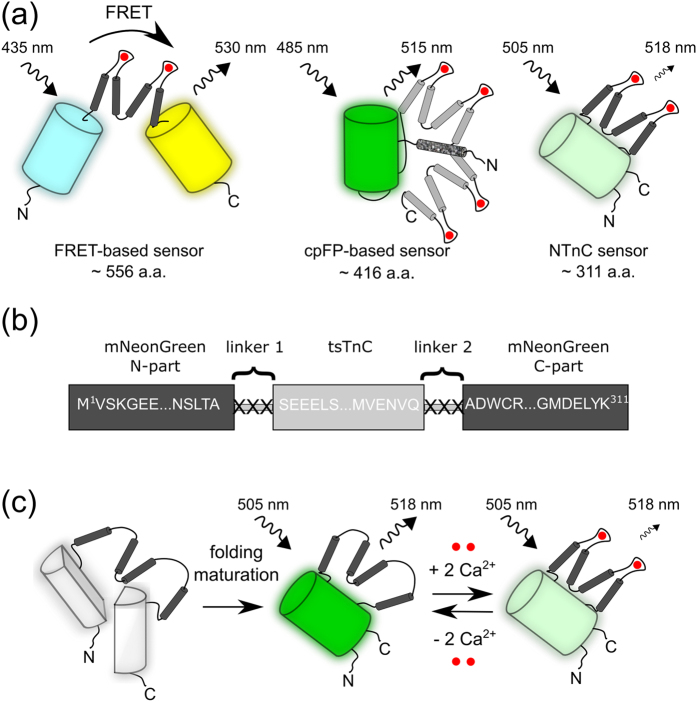
Schematic representation of the different designs of GECIs, composition of the original library, and suggested stages of NTnC Ca^2+^ indicator function. (**a**) Schematic representation of FRET-based, cpFP-based, and NTnC sensor families in the Ca^2+^-bound state. FPs are shown as cylinders, and tsTnC, CaM and M13-peptide are shown in dark grey, light grey, and speckled grey, respectively. (**b**) The original library for NTnC consisted of the sensory C-terminal minimal domain of TnC (tsTnC) inserted into the mNeonGreen fluorescent protein between residues 145 and 146, with randomized linkers located between the fluorescent and sensory components. (**c**) Schematic representation of the NTnC indicator formation and functioning. The mNeonGreen component is shown as a cylinder, which reversibly quenches upon binding with two Ca^2+^ ions (red dots).

To design a small sensor with two binding sites for calcium ions, we selected as the sensory part the C-terminal minimal domain of TnC, which is expressed in the swim bladder and white muscles of *O. tau* (tsTnC). This domain has been previously employed in the Twitch family of FRET sensors^3^. For the fluorescent component, we used green FP mNeonGreen, which is brighter than the other FPs of its spectral class^9^. We inserted the sensory part between residues 145 and 146 of mNeonGreen and randomized both of the 3-amino-acid-long linkers between the fluorescent and sensory components (Fig. 1). To identify sensor variants with the highest ΔF/F response, we implemented a hierarchical two-step screening strategy. First, we performed imaging on a library of sensors with randomized linkers targeted to the *E. coli* periplasm on Petri dishes with media supplemented with Ca^2+^ ions and compared the resulting fluorescent signals before and after treatment with a buffer containing EDTA. Second, clones with the best Ca^2+^/Ca^2+^-free ratio identified in the first screen were analyzed in bacterial suspension. The best mutants found after screening in a bacterial library with randomized linkers were further subjected to several rounds of random mutagenesis and selection. During each round, we screened approximately 20,000–40,000 colonies to identify variants with the largest Ca^2+^-dependent changes in green fluorescence. Finally, the variant with the best performance, named NTnC, had 18 mutations relative to the original library (Supplementary Fig. 1), with 9 and 3 mutations located in the fluorescent and sensory sections, respectively. In the fluorescent component, 4 mutations were internal and buried in the β-barrel, and 5 others were external. None of the external mutations resided in the A/B or A/C dimerizing interfaces^9^; these external mutations may be necessary for efficient protein folding and interaction with the sensory unit. Internal mutations should act primarily to adjust the fluorescent properties of the chromophore. Among the internal mutations, only the F240L mutation (Supplementary Fig. 1), corresponding to position 165 in GFP (Supplementary Fig. 2), was located close to the Tyr69 ring of the chromophore, judging from the crystal structure of EGFP^10^. The other 3 internal mutations were found outside of the immediate surroundings of the chromophore and are unlikely to significantly affect chromophore fluorescence. In the sensory component, an N165D mutation (corresponding to position 17 in the tsTnC minimal domain^3^) was located in the Ca^2+^-chelating EF hand 3 of TnC and should act to enhance Ca^2+^ binding^3^. The other 2 mutations were also distributed over the EF hand 3, suggesting that this motif may be a major contributor to the features of the sensor.

In an attempt to improve the dynamic range of NTnC, we prepared four additional libraries with shortened linkers (Supplementary Fig. 3). Screening of these libraries did not reveal mutants with ΔF/F responses higher than that of NTnC.

### *In vitro* characterization of purified NTnC

**Figure 2:**
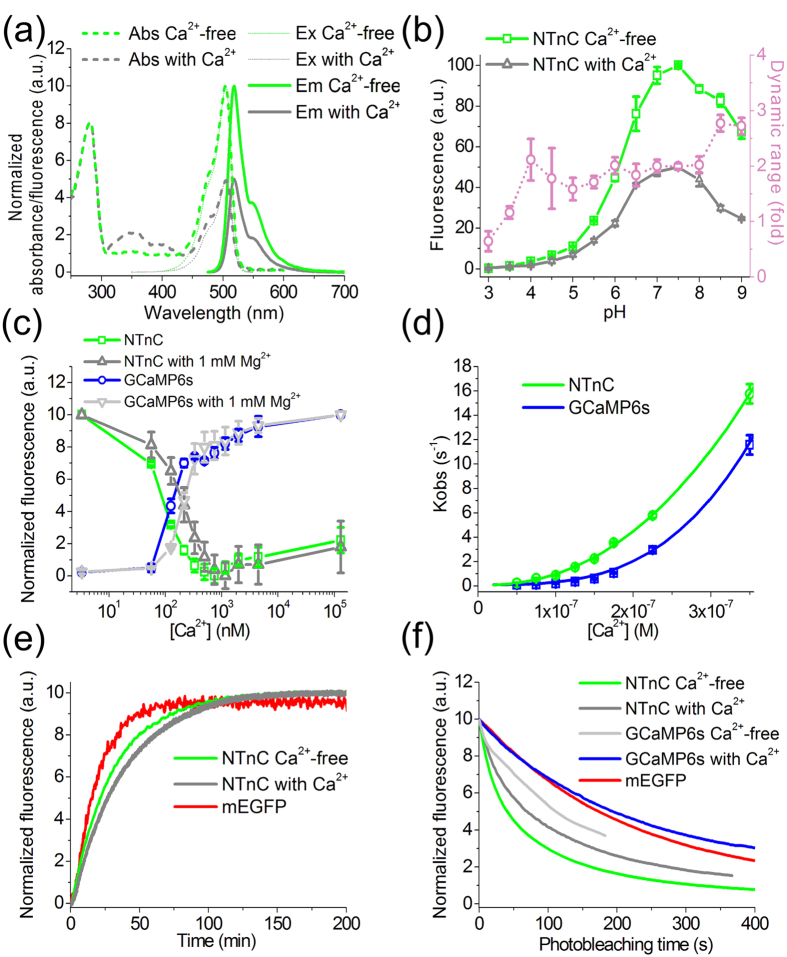
*In vitro* properties of purified NTnC protein. **(a)** Absorbance, excitation and emission spectra of NTnC in Ca^2+^-free and Ca^2+^-bound states. **(b)** Intensity and dynamic range of NTnC as a function of pH. The dynamic range (fold) at each pH value was determined as the ratio of NTnC fluorescence intensity in the absence of Ca^2+^ to that in the presence of Ca^2+^. **(c)** Ca^2+^ titration curves for NTnC and GCaMP6s in the absence or presence of 1 mM MgCl_2_. **(d)** Observed Ca^2+^ association rates at moderate Ca^2+^ concentrations (in the range of 0–350 nM) overlaid with the fitting curves (k_obs_ = k_on_ × [Ca^2+^]^n^ + k_off_, see Table 1 for the fitting parameters k_on_ and n). **(e)** Maturation curves for NTnC in the Ca^2+^-free (green line) and Ca^2+^-bound (grey line) states and for mEGFP (red line). **(f)** Photobleaching curves for NTnC and GCaMP6s in Ca^2+^-free and Ca^2+^-bound states and for mEGFP. Error represents the standard error of the estimate for the average of three records.

The *in vitro* characteristics of the purified calcium sensor NTnC are summarized in Table 1. NTnC exhibited green fluorescence with excitation and emission peaks at 505 and 518 nm, respectively (Fig. 2a). According to its brightness in the Ca^2+^-free state, NTnC outperformed the control GCaMP6s in its bright, Ca^2+^-saturated state by 1.5-fold. Enhanced brightness of NTnC supports the notion that the choice of fluorescent template (in our case, bright mNeonGreen protein) is a critical step that strongly pre-determines the properties of the engineered protein^11^.

NTnC binding of calcium ions was accompanied by minor changes in the fluorescence quantum yield of its chromophore compared with the 2-fold fluorescence response (Table 1). Upon binding of Ca^2+^ ions, we observed a 2-fold reduction in absorbance at 505 nm and increases in absorbance for the sensor at 350 and 400 nm (Fig. 2a). These observations suggest that binding of NTnC to Ca^2+^ ions is accompanied by transition from one form of the chromophore (with absorbance at 505 nm) into at least one other form (with absorbances at 350 and 400 nm). The opposite transition is observed upon dissociation of NTnC from Ca^2+^ ions. The 350- and 400-nm absorbing forms have fluorescence maxima at 416 and 518 nm, respectively (data not shown). Acidification and alkalization of NTnC resulted in major and slight increases in its absorbances at 400 and 350 nm, respectively (Supplementary Fig. 4). The 350- and 400-nm absorbing forms can be attributed to the differently protonated GFP chromophore, similar to the proteins Sirius^6^ and T-Sapphire^12^. In the Ca^2+^-bound state, NTnC produced ~400- and 130-fold lower fluorescence signal when excited at 350 and 400 nm, respectively, compared with that at the 505-nm excitation. Thus, the brightness levels of the 350- and 400-nm absorbing forms are negligible when compared with that of the main 505 nm absorbing form. Importantly, absorbance at 505 nm is reduced upon binding Ca^2+^, thus demonstrating an inverted fluorescence response to Ca^2+^.

In the Ca^2+^-free and Ca^2+^-bound states, NTnC exhibited practically identical p*K*a values of 6.1 (Fig. 2b). As a result, its fluorescence response essentially did not change over the broad pH range of 4–8. Additionally, the fluorescence of NTnC in both states did not depend on pH within the range of 7–8. In contrast to NTnC, GCaMP6s had significantly different p*K*a values in the Ca^2+^-free (p*K*a ≥ 9.6) and Ca^2+^-bound states (p*K*a = 6.16), resulting in a pronounced pH-dependence of its ΔF/F response within the pH range of 5–8 (Supplementary Fig. 5). GCaMP6s fluorescence in the Ca^2+^-free state is strongly dependent on pH. The increased pH stability of NTnC in both states makes NTnC more specific to changes in Ca^2+^ concentrations, with only negligible effects of pH. Indeed, commonly used green GECIs, such as G-GECOs^13^ and GCaMPs^14^, have large p*K*a values in their Ca^2+^-free states that should result in their sensitivity to pH changes. However, the impact of such pH changes has not been estimated for these GECIs *in vivo*.

According to equilibrium binding titrations, NTnC demonstrated a 1.7-fold higher binding affinity for Ca^2+^ ions than GCaMP6s (Fig. 2c and Table 1). Its equilibrium K_d_ value was 1.8- to 3-fold smaller than the K_d_ value for the FRET sensors Twitch-1/2/3, which are based on the same TnC domain^3^. The enhanced binding affinity of NTnC to Ca^2+^ ions may be attributed to the N165D mutation (corresponding to position 17 in the minimal domain tsTnC^3^), which is located in the Ca^2+^-chelating loop. The small increases in NTnC fluorescence at high Ca^2+^ concentrations in the range of 10^3^–10^5^ nM may be associated with some interactions at the binding sites for Ca^2+^ ions. This possibility is supported by the absence of such fluorescence increases for the NTnC/166D+/202D+ mutant with Asp amino acid insertions at positions 166 and 202, in which the affinity to Ca^2+^ ions is abolished (Supplementary Fig. 6a). We cannot exclude the possibility that at high Ca^2+^ concentrations, admixtures of other metal ions such as Mg^2+^ may compete with Ca^2+^ ions for binding sites. We also do not know whether this is the case in living cells. In any case, the fluorescence increase of NTnC at high Ca^2+^ ion concentrations in living cells may only slightly decrease its response amplitude (by 20%). Equilibrium Hill coefficients for NTnC and GCaMP6s indicators effectively coincided with the presence of two and four Ca^2+^-binding sites in the respective TnC and CaM sensory components.

Equilibrium binding titration of NTnC with magnesium ions (Mg^2+^) showed that the NTnC fluorescence did not noticeably change up to a 50 mM concentration of Mg^2+^ (Supplementary Fig. 7). In the presence of 1 mM Mg^2+^, purified NTnC and GCaMP6s demonstrated similar increases in K_d_ values of 2.3- and 1.6-fold, respectively (Fig. 2c and Table 1). At 1 mM Mg^2+^, a concentration that resembles that in neuronal cytoplasm, NTnC demonstrated the same ΔF/F response of 2.0 as in the absence of Mg^2+^ ions. These data indicate that Mg^2+^ ions do not hinder the binding of NTnC to Ca^2+^ ions.

Ca^2+^ association and dissociation kinetics were studied using stopped-flow fluorimetry. Analysis of the dependence of observed Ca^2+^ association rates on Ca^2+^ concentrations (in the range of 0–350 nM; Supplementary Fig. 8a and Fig. 2d) allowed us to estimate Hill coefficients and to reassess the dissociation constants. K_d_^kin^ values for NTnC and GCaMP6s indicators are rather similar to those determined from the equilibrium studies (Table 1). A lower Hill coefficient for NTnC than for GCaMP6s is consistent with the notion of fewer Ca^2+^-binding sites in the C-terminal domain of TnC than in CaM. Analysis of the observed Ca^2+^ association rates at higher Ca^2+^ concentrations (>350 nM) revealed reduced cooperativity and an approximately 5-fold lower onset limiting rate (k^onset^_limit_) of NTnC compared with GCaMP6s (Supplementary Fig. 9), which might reflect the NTnC slower rate resolution of fluorescence changes. The half-time of NTnC-Ca^2+^ dissociation was 3-fold higher than that for the GCaMP6s-Ca^2+^ complex (t_1/2_^NTnC^ = 3 s, t_1/2_^GCaMP6s^ = 1 s, Supplementary Fig. 8b), which might account for the improved sensitivity of NTnC at low Ca^2+^ concentrations. The stopped-flow kinetics data indicate that NTnC responds more slowly to Ca^2+^ than does GCaMP6s, which may hinder fast kinetics studies *in vivo*; further protein-engineering efforts are needed to improve the kinetics of NTnC Ca^2+^-response.

At 37 °C, NTnC matured in the Ca^2+^-free and Ca^2+^-bound states 1.6- and 2-fold slower than mEGFP, respectively (Fig. 2e). Because of poor expression levels of GCaMP6s, we could not fully characterize its maturation rate.

Under a wide-field microscope equipped with a metal halide lamp, NTnC sensors in the Ca^2+^-free and Ca^2+^-bound states photobleached 3.7- and 2.7-fold faster, respectively, than GCaMP6s (Fig. 2f).

In the presence of 5 mM Ca^2+^ and in the absence of Ca^2+^, both purified NTnC and control GCaMP6s sensors eluted on size-exclusion chromatography as monomers (Supplementary Fig. 10); note that monomeric proteins are usually preferable in terms of cytotoxicity^15^.

### Specific response of the developed NTnC calcium sensor to the addition of external calcium ions in HeLa Kyoto mammalian cells

**Figure 3:**
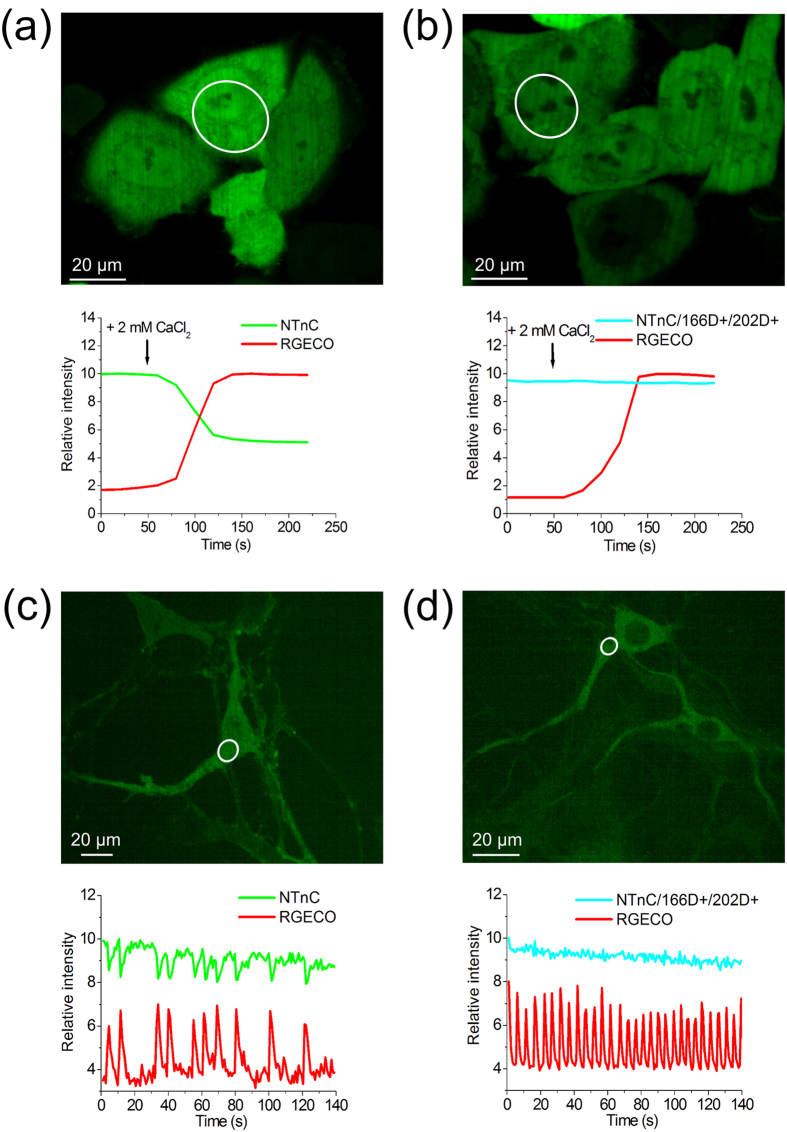
Response of NTnC to variations in Ca^2+^ concentration in HeLa cells and neuronal cultures. **(a)** HeLa Kyoto cells co-expressing NTnC and R-GECO1. The graph illustrates green and red fluorescence changes in response to the addition of 2 mM CaCl_2_ and 5 μM ionomycin. **(b)** Co-expression of the NTnC/166D+/202D+ mutant, which has inhibited binding affinity, together with R-GECO1 in HeLa Kyoto cells. The graph shows the changes in green and red fluorescence as a result of the addition of 2 mM CaCl_2_ and 5 μM ionomycin. R-GECO1 is co-expressed as a control and confirms the increase in Ca^2+^ concentration. **(c**) Dissociated neuronal culture co-expressing NTnC and R-GECO1 sensors. The graph shows the green and red fluorescence changes of the NTnC and R-GECO1 indicators as a result of spontaneous neuronal activity. **(d)** Dissociated neuronal culture co-expressing the NTnC/166D+/202D+ mutant and R-GECO1. Graph shows green and red fluorescence changes of the NTnC/166D+/202D+ mutant and R-GECO1 as a result of spontaneous neuronal activity. (**a**–**d**) For cellular images, the red channel is not shown. The graphs illustrate changes in green and red fluorescence in the areas indicated with white circles.

To characterize our NTnC sensor in mammalian cells, we expressed it in the cytoplasm of HeLa Kyoto cells. After the addition of 2 mM external CaCl_2_ with ionomycin, the sensor demonstrated a 2-fold maximal change of its fluorescence in approximately 2 min (Fig. 3a and Supplementary Fig. 11a). When coexpressed in the same cells, the red calcium indicator R-GECO1 and NTnC demonstrated similar dynamics (Fig. 3b). To confirm that the observed fluorescence changes of NTnC were specific to Ca^2+^ concentration changes, we generated the NTnC/166D+/202D+ mutant of NTnC, in which the binding affinity to Ca^2+^ ions was abolished. With the free Ca^2+^ ion concentration in the range of 0–39 μM, NTnC/166D+/202D+ did not bind Ca^2+^ ions and exhibited a pH dependence of fluorescence similar to that of the original NTnC sensor (Supplementary Fig. 6). When expressed in HeLa Kyoto cells, NTnC/166D+/202D+ did not show changes in its green fluorescence upon the addition of 2 mM CaCl_2_ and ionomycin mixture (Fig. 3b). At the same time, R-GECO1 co-expressed with NTnC/166D+/202D+ in the same cell demonstrated the expected response to Ca^2+^ concentration changes. Our results indicate that, in mammalian cells, the NTnC sensor undergoes specific changes in its fluorescence in response to variations in Ca^2+^ concentration; these results also suggest that NTnC/166D+/202D+ mutant protein can be used as a control to estimate the impact of conditions that do not reflect changes in Ca^2+^ ions but could influence NTnC fluorescence (e.g., changes in pH).

### Visualization of spontaneous neuronal activity in dissociated culture using NTnC

We next sought to demonstrate functionality of the NTnC in neurons in dissociated neuronal cultures during their spontaneous activity. To this end, we transduced neuronal cultures with recombinant AAV (rAAV) carrying various calcium indicators under control of the CAG promoter. We successfully registered spontaneous activity of neurons in two- to three-week-old cultures, manifested as a 20% decrease in fluorescence for NTnC and 60% increases for GCaMP6s and R-GECO1 when measured in the green or red channels (Fig. 3c and Supplementary Fig. 11b and Supplementary Table 1). The decreased ΔF/F response of the NTnC calcium sensor observed during spontaneous activity of neuronal cultures, compared with those of pure protein and expression in HeLa cells, could be partially explained by the relatively high concentration of free Ca^2+^ in resting neurons, which has been estimated as 50–100 nM (increasing 5-fold to 100-fold in active neurons)^16^,^17^. Indeed, as is evident from the Ca^2+^ titration curve for NTnC, at concentrations of free Ca^2+^ of 50–100 nM, 30–60% of the sensor is bound to Ca^2+^ ions (Fig. 2c). The dynamics of NTnC were effectively identical to those of R-GECO1 and GCaMP6s. The NTnC/166D+/202D+ mutant with blocked Ca^2+^ affinity did not show any changes in fluorescence, despite ongoing spontaneous neuronal activity confirmed by R-GECO1 (Fig. 3d). Overall, these data indicate that during spontaneous activity of neurons, NTnC specifically reports changes in the Ca^2+^ concentration through a drop in green fluorescence.

### Characterization of NTnC in dissociated neuronal culture with whole-cell patch clamp

**Figure 4:**
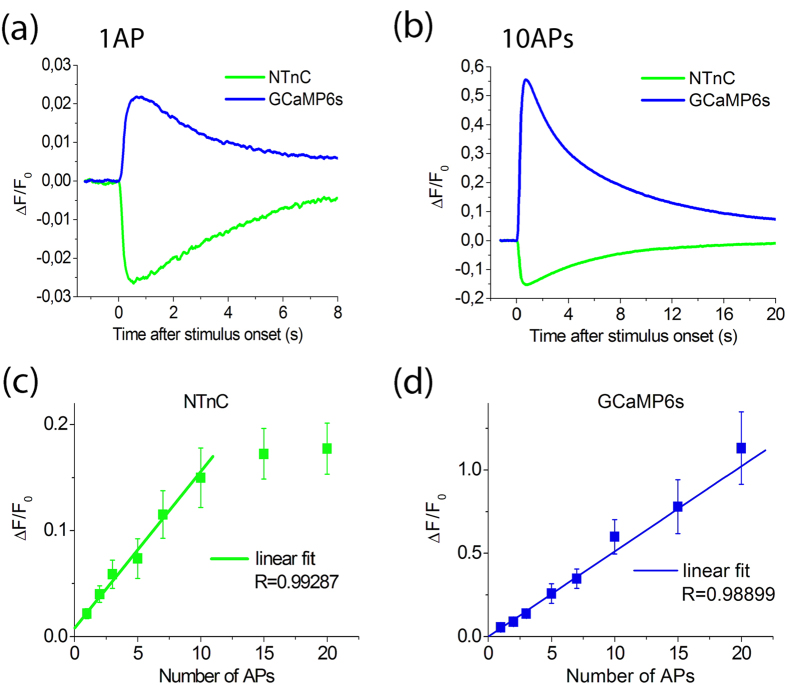
Fluorescence changes in response to intracellularly induced APs in cultured neurons expressing the indicators NTnC and GCaMP6s. (**a**) Response to a single AP. (**b**) Response to a train of 10 APs at 50 Hz. All responses were averaged across all recorded neurons in different wells. Note similar signal amplitudes and response kinetics induced by single APs in NTnC and GCaMP6s cells. (**c**,**d**) Dependence of the amplitudes of responses induced by different numbers of APs in neurons expressing NTnC (N = 6) and GCaMP6s (N = 7). Note that in the range from 1 to 10 APs, dependence is linear for both sensors. The linear regression shown in the figure was calculated for the 1–10 APs subset for NTnC and for the whole data range (1–20 APs) for GCaMP6s. Values are shown as the means ± SEM.

To further characterize the performance of NTnC in neurons, we compared responses of NTnC and GCaMP6s expressed in neuronal cultures using whole-cell patch recording. In one series of these experiments, we measured the fluorescent changes in cells in response to 1 and 10 action potentials (APs) induced intracellularly at a 50-Hz frequency. Both indicators demonstrated fast and reliable changes in fluorescence levels for both 1 and 10 APs (Fig. 4a,b and Supplementary Fig. 12). As expected, stimulation of GCaMP6s-expressing neurons induced an increase in fluorescence level, while NTnC-expressing cells showed a drop in fluorescence in response to intracellular stimulation. The kinetics of the Ca^2+^ responses (rise and decay half-times) in neurons expressing NTnC and GCaMP6s did not show any significant differences (Table 2). The signal-to-noise ratio (SNR) and ΔF/F also demonstrated very similar values and did not differ significantly in response to 1 AP for the two indicators. However, fluorescence changes induced in GCaMP6s-expressing neurons by a train of 10 APs showed 3.7- and 5-fold greater ΔF/F and SNR, respectively, than those observed in the NTnC-expressing neurons (p < 0.01 for both parameters, Mann-Whitney Rank Sum Test). These differences may reflect the different dynamic ranges for the two sensors. In neurons, NTnC and GCaMP6s showed similar sensitivity to a single AP, despite the lower dynamic range of NTnC vs. GCaMP6s. The limited dynamic range of NTnC is slightly compensated for by the 1.2-fold higher affinity of NTnC to Ca^2+^ ions, compared with that of GCaMP6s (Table 1). In contrast to GCaMP6s, NTnC has a linear and larger response to low Ca^2+^ concentration changes within the range of 0–200 nM (Supplementary Fig. 13). This difference could be explained by the reduced number of Ca^2+^-binding sites in NTnC^2^. The linear response of NTnC fluorescence at low Ca^2+^ concentration changes may additionally enhance the sensitivity of NTnC to a single AP.

In a separate series of experiments, we estimated the linearity of responses elicited by different numbers of APs in cells expressing NTnC and GCaMP6s. We recorded responses to 1, 2, 3, 5, 7, 10, 15, and 20 APs induced with a frequency of 50 Hz. Response kinetics were not analyzed in this series. In the range of 1 to 10 APs, both indicators showed linear dependence of the maximal ΔF/F on the number of APs (Fig. 4c,d). However, with more than 10 APs, the dependence of the response amplitude on the number of action potentials in NTnC-expressing neurons showed pronounced saturation, while in GCaMP6s-expressing cells, this dependence remained effectively linear.

Thus, in neurons, NTnC robustly and linearly responded to stimulation with 1–10 APs and demonstrated Ca^2+^ ion association-dissociation kinetics similar to the GCaMP6s sensor.

### Electrophysiological characteristics of NTnC-expressing neuronal cultures on MEAs

To determine whether NTnC expression in neurons may have adverse effects on neuronal function, we examined the electrophysiological properties of neuronal cultures transduced with NTnC or GCaMP6s and of control non-transduced cultures, all plated on MEAs. To evaluate the impact of NTnC or GCaMP6s expression on spontaneous electrical activity of neuronal cultures, we determined spiking rates at day *in vitro* (DIV) 9, 13, and 16 (Supplementary Fig. 14). As expected^18^, the spiking rates increased ~3-fold from DIV 9 to DIV 13, reaching a plateau. At each time point, these rates did not differ among the three types of cultures, non-transduced, NTnC-transduced, and GCaMP6s-transduced.

Next, we sought to reveal potential differences between the NTnC- or GCaMP6s-transduced and control neuronal cultures by examining additional parameters, such as time intervals between bursts and the number of spikes in bursts. For each culture type, we determined summarized distributions of both the time intervals between bursts and the numbers of spikes in bursts at each day tested (Supplementary Figs 15a–c and 16a–c).

The median interburst time intervals, as calculated from the summarized distributions, did not differ among the three types of cultures at each time point (taking into account the spread of median values for individual cultures; Supplementary Fig. 15d and Supplementary Table 2). By DIV 13, the time intervals became 1.7- to 4-fold shorter and subsequently remained stable.

The median number of spikes in bursts increased 2.5- to 3.3-fold by DIV 13 in all three types of cultures (Supplementary Fig. 16d and Supplementary Table 2). At DIV 16, control non-transduced cultures additionally increased the number of spikes in bursts by 1.6-fold, whereas in both NTnC- and GCaMP6s-transduced cultures, this parameter remained the same and did not differ between the two types of cultures. The difference between the transduced and control cultures may reflect a negative impact of viral transduction on the electrophysiological properties of neuronal cultures. Notably, all electrical characteristics of NTnC- and GCaMP6s-transduced cultures were similar. Taken together, our results suggest that the electrophysiological properties of cultured neurons are similarly affected by the expression of NTnC or GCaMP6s sensors.

### *In vivo* two-photon imaging of neuronal activity in the visual cortex of awake mice using NTnC

**Figure 5:**
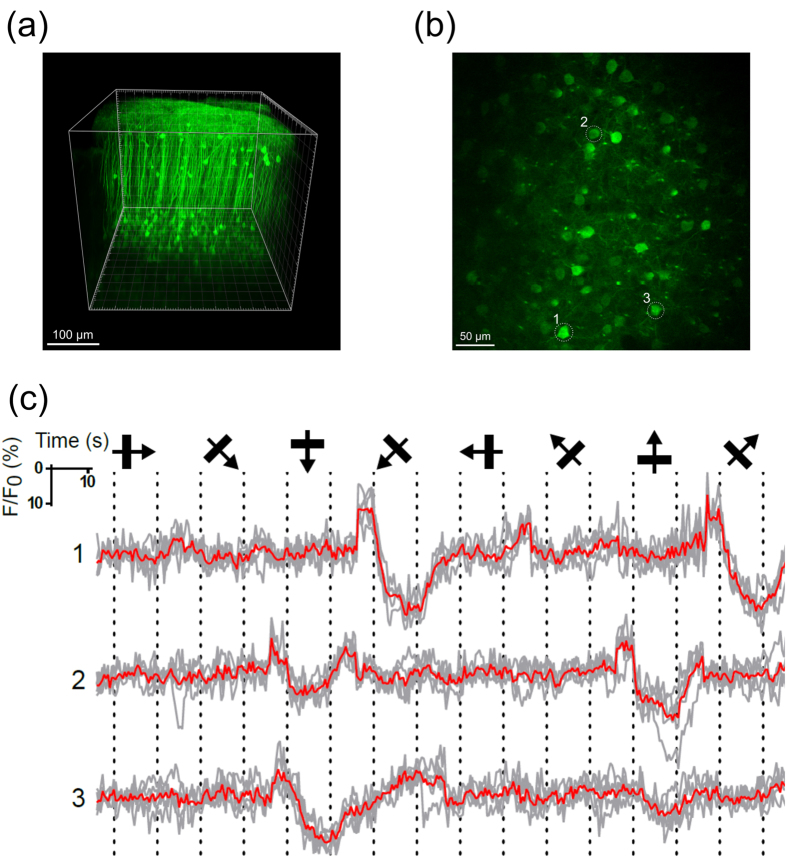
*In vivo* complex visual stimuli evoked neuronal Ca^2+^ activity in the mouse cortex as visualized with the calcium sensor NTnC and two-photon microscopy. (**a**) Representative image of a 3D volume reconstruction of the mouse V1 at 11 weeks following stereotactic injection of rAAV (AAV-*CAG*-NTnC) viral particles. (**b**) Two-photon image of cells in a mouse visual cortex visualized with the calcium indicator NTnC, imaged *in vivo* at 460 μm below the pial surface. (**c**) Time-courses of cells 1–3, as marked in (**b**). Average Ca^2+^ traces (ΔF/F_0_) from three neurons during stimulation with the presentation of drifting gratings (eight directions, five repetitions). The directions of the drifting gratings are shown; dashed lines show drift onset and offset, grey – individual trial, red – mean signal over all five repetitions.

To test the capacity of NTnC for *in vivo* registration of neuronal activity, we used two-photon microscopy to visualize neuronal Ca^2+^ activity in the visual cortex of awake mice with fixed heads in response to complex visual stimuli represented by standard gratings moving in different orientations. We transduced layer 2/3 of the primary visual cortex of mice with rAAV particles expressing NTnC under the control of the CAG promoter (AAV-*CAG*-NTnC). The cranial window was installed simultaneously with the viral transduction. On days 60–65 after rAAV infection, we imaged the transduced brain region beneath the optical window using a two-photon microscope. In the 3D reconstruction, we observed NTnC expression that was evenly distributed across the pyramidal neuronal cell bodies of layer 2/3 and their axons and dendrites (Fig. 5a). To confirm the capability of NTnC to reveal visual stimulus-evoked neuronal activity, we performed time-lapse imaging of the region shown in Fig. 5b during presentation of gratings drifting in 8 different orientations to the contralateral eye. Fluorescence changes in the somas of neurons from this region showed orientation-specific responses that were reproducible across five trials (Fig. 5c). Presentation of visual stimuli produced activation in 13 ± 4% of neurons (estimated from 4 fields of view); this fraction of responding neurons detected with NTnC was ~5-fold lower than for GCaMP6s^14^. Averaged neuronal activity lasted for the entire stimulus presentation and was evident in both directions at a specific orientation. The same orientation preference for neurons in a similar experimental model was earlier shown for the GCaMP6s sensor^14^. Thus, these results indicate that NTnC can reveal stimulus-evoked neuronal activity *in vivo*.

### *In vivo* recording of neuronal activity in the visual cortex of freely moving and anesthetized mice using NTnC and an nVista miniaturized microscope

**Figure 6:**
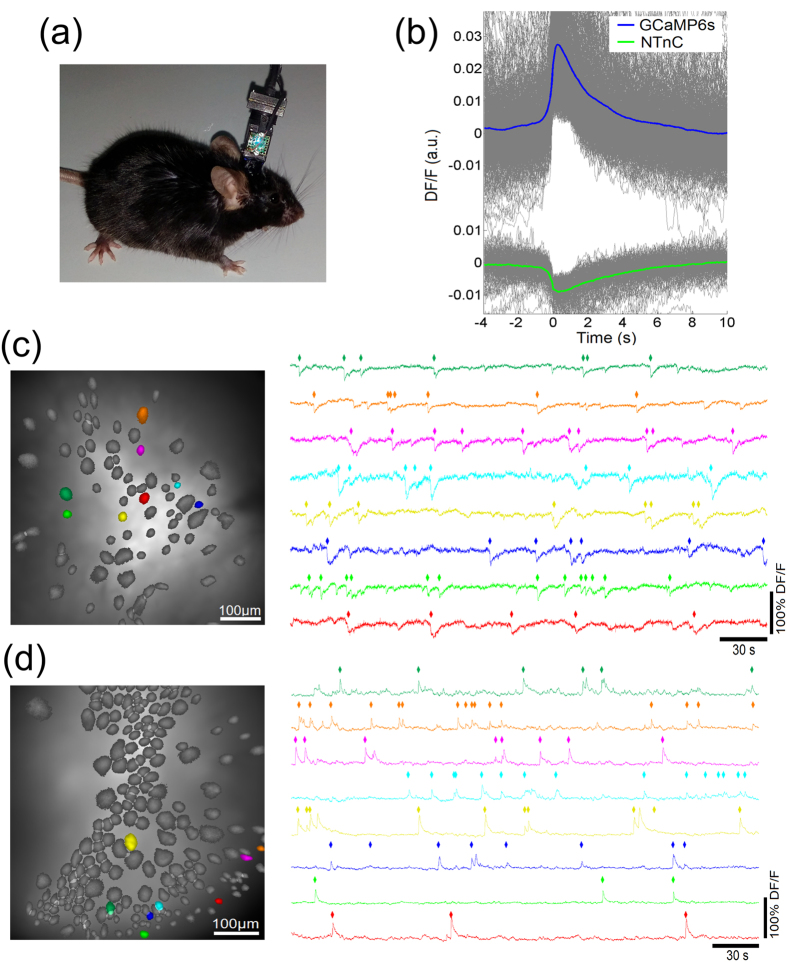
*In vivo* neuronal Ca^2+^ activity in freely behaving mice visualized with the calcium indicators NTnC and GCaMP6s and an nVista HD system. (**a**) Photo of an nVista HD miniature head-mounted microscope attached to a mouse’s head. (**b**) Mean spikes in the calcium indicators NTnC and GCaMP6s; spikes were aligned at the moment of 4 MAD threshold crossing (0 s); only single spikes were considered, i.e., a spike was taken into account only if there were no other spikes for 10 s after and 4 s before it. (**c**) Spatial filters and sample traces obtained from a 5-min imaging session with a freely behaving mouse expressing NTnC; rhombi over traces denote spikes that were counted as 4 MAD threshold crossings. (**d**) Spatial filters and sample traces obtained from a 5-min imaging session with a freely behaving mouse expressing GCaMP6s. The sensors NTnC and GCaMP6s were delivered to brain cortices with rAAV (AAV-*CAG*-NTnC and AAV-*CAG*-GCaMP6s) particles.

To examine whether NTnC can be used for *in vivo* recording of neuronal activity with an nVista head-mounted miniaturized microscope, we attempted to visualize neuronal Ca^2+^ activity in the visual cortex of anesthetized mice in response to simple (pulses of light) and complex (moving grating) visual stimuli and in freely moving mice during context exploration. We installed the nVista microscope over layer 2/3 of the visual cortex of mice transduced with rAAV particles carrying NTnC or control GCaMP6s green calcium indicators under the control of the CAG promoter (Fig. 6a). During the installation procedure, the Ca^2+^ activity of neurons was dramatically reduced due to the anesthesia; notably, the inverted phenotype of NTnC facilitated identifying and focusing on the neurons in the transduced layer. To obtain non-saturating signals for NTnC, we set the LED power of the nVista microscope to ~25% (compared with ~90% power for GCaMP6s). We could not detect reproducible stimulus-evoked neuronal Ca^2+^ activity in anesthetized mice in response to either simple or complex visual stimuli (Supplementary Figs 17 and 18 and Supplementary Results).

For freely moving mice, which were placed in the cage to explore, we successfully imaged and assigned 79 and 199 cells to NTnC and GCaMP6s, respectively (Fig. 6 and Supplementary videos 1 and 2). As expected, NTnC demonstrated an inverted phenotype, and its averaged single anti-spikes had rise and decay half-times of 0.3 and 2.5 sec, respectively, slightly larger than those for GCaMP6s (Fig. 6b). Identified cells and example traces of activity for fluorescent neurons are presented in Fig. 6c,d. Based on the fluorescence changes characteristic of neuronal activity, we found that 72 and 94% of assigned cells were active for NTnC and GCaMP6s-expressing neurons, respectively (Supplementary Table 3). On average, NTnC-expressing neurons demonstrated 1.4-fold fewer spikes per second compared with GCaMP6s-expressing neurons. Additionally, compared with GCaMP6s, the averaged peak ΔF/F value for NTnC was 2.5-fold lower, while the SNR value for NTnC was similar to that for the GCaMP6s sensor, likely because of the enhanced brightness of NTnC. Thus, we were able to use NTnC to visualize neuronal dynamics in an *in vivo* model with freely moving mice using an nVista miniaturized microscope.

### Summary

Using directed molecular evolution in bacteria, we developed a new genetically encoded green calcium sensor, NTnC, with a novel design that combines the benefits of FRET-based sensors of the Twitch family and cpFP-based sensors. We have characterized the main features of this new intensiometric sensor both *in vitro* and *in vivo*. NTnC has high brightness due to the high intrinsic brightness of its parental protein, mNeonGreen. It also maintains other beneficial characteristics of the parental protein, such as pH stability and monomeric behavior. NTnC has an inverted phenotype, i.e., it reduces its fluorescence upon the binding of Ca^2+^ ions. Compared with other commonly used sensors, it is ~100 amino acids smaller in size and has half the number of Ca^2+^-binding sites (Fig. 1a). The insertion version of the mNeonGreen protein that we have developed and its use for a new design of a GECI may help to improve the brightness of other single-fluorophore-based sensors and may assist in the generation of other mNeonGreen-based sensors.

We have expressed NTnC in mammalian and neuronal cells and characterized its features *in vivo*. NTnC could reliably report variations in Ca^2+^ ion levels induced by ionomycin in mammalian cells and by spontaneous activity in dissociated neuronal cultures. Specific responses of the NTnC indicator to calcium ions have been confirmed with a control mutant, NTnC/166D+/202D+. The availability of such a control for NTnC opens up possibilities for further research to distinguish the impact of Ca^2+^ from those of other factors (such as pH) that may affect the fluorescence of the indicator.

Using whole-cell patch clamp recording, we have revealed similar kinetics of Ca^2+^ responses in neurons expressing NTnC and GCaMP6s. We have found that NTnC shows sensitivity to single APs similar to that of GCaMP6s. This sensitivity correlates with the higher affinity of NTnC to Ca^2+^ ions and with its linear response at low Ca^2+^ concentration changes, which is due to the decreased number of Ca^2+^-binding sites. Furthermore, our results indicate similar impacts of NTnC and GCaMP6s expression on the electrophysiological features of the MEA-plated neurons.

Finally, we have explored the potential of NTnC for *in vivo* applications. NTnC has revealed stimulus-evoked neuronal calcium ion activity *in vivo* in the visual cortex of awake mice with the help of two-photon microscopy. NTnC can also successfully visualize neuronal activity *in vivo* in the visual cortex of freely moving mice using an nVista head-mounted miniature microscope (notably, the high brightness and inverted phenotype of NTnC facilitated the installation procedure preceding *in vivo* imaging). During *in vivo* experiments, NTnC revealed less neuronal calcium ion activity than did GCaMP6s, which might be related to the limited ΔF/F dynamic range of NTnC.

Importantly, the enhanced baseline brightness and inverted fluorescence responses of NTnC facilitate the identification of neurons at low resting concentrations of calcium ions and, thus, may be valuable for *in vivo* applications where background fluorescence makes detection of such cells problematic. We expect that further exploration of NTnC-like designs, with the aim of enhancing its ΔF/F dynamic range, may result in sensors with performance levels similar or superior to those of GECIs with conventional designs.

## Materials and Methods

### Mutagenesis and library screening

Primary construction of sensors and directed saturated mutagenesis of linkers between fluorescent and sensory parts were accomplished using polymerase chain reaction (PCR) with overlapping fragments^19^. For PCR amplification, we used a С1000 Touch Thermal Cycler (Bio-Rad, USA). Random mutations were introduced over the whole length of the sensor gene using PCR in the presence of manganese ions with conditions to achieve 2–3 random mutations per 1000 bp (according to the Diversify PCR Random Mutagenesis Kit User Manual, Clontech, USA). Alternatively, random mutagenesis of the sensor was performed with a GeneMorph II Random Mutagenesis Kit (Agilent Technologies, USA).

Further, we cloned genes for sensors using the BglII/EcoRI restriction sites of the pBAD/His-TorA plasmid encoding the TorA signal sequence, which is necessary for the transport of sensors into the periplasmic space of bacteria, and transformed these plasmids into bacteria. To that end, PCR products were purified in 1% agarose gels and extracted using PCR purification and a gel extraction kit (Evrogen, Russia). Afterwards, plasmids and PCR digests were ligated. Ligation mixes were further purified via PCR purification and using a gel extraction kit (Evrogen, Russia) and were transformed into electrocompetent BW25113 bacteria using electroporation.

Screening of bacterial libraries was performed sequentially on Petri dishes, bacterial suspensions in 96-well plate format, and purified proteins (Supplementary Methods).

### Protein purification and characterization

The genes for protein expression were cloned into the pBAD/HisB vector (Invitrogen, USA) at the BglII/EcoRI restriction sites, and the resulting plasmids were transformed into BW25113 bacteria. The bacterial cultures were grown in LB medium supplemented with 0.02% arabinose and 100 μg/ml ampicillin overnight at 37 °C and 220 rpm. The cultures were then centrifuged at 4648 g for 10 min, and the cell pellets were resuspended in PBS at pH 7.4 with 300 mM NaCl and lysed by sonication on ice. The recombinant proteins were purified using Ni-NTA resin (Qiagen, USA), followed by dialysis for 12–16 h against buffer solutions (10 mM Tris-HCl, 100 mM KCl, pH 7.2, with either 10 mM EDTA or 10 mM CaCl_2_ or without EDTA and CaCl_2_). The absorbance values and excitation and emission spectra were measured with a CM2203 spectrofluorometer (Solar, Belarus).

Chromophore extinction coefficients for purified NTnC in the Ca^2+^-free and Ca^2+^-saturated states were measured in buffer (10 mM Tris-HCl, 100 mM KCl, pH 7.2) supplemented with either 10 mM EDTA or 10 mM CaCl_2_, respectively, by alkaline denaturation with 1 M NaOH and using extinction coefficients for GFP-like chromophores equal to 44,000 M^**−**1^ cm^**−**1^ in 1 M NaOH^20^.

For quantum yield determination, the integrated fluorescence values of purified NTnC and GCaMP6s in the Ca^2+^-free and Ca^2+^ saturated states were measured in buffer (10 mM Tris-HCl, 100 mM KCl, pH 7.2) supplemented with either 10 mM EDTA or 10 mM CaCl_2_, respectively, and were compared with equally absorbing GCaMP6s in the saturated state (quantum yield of 0.61) or mTagBFP2 (quantum yield of 0.64), as previously reported^21^.

Purified NTnC (5 mg/ml) and GCaMP6s (2 mg/ml) proteins were diluted 1:10,000 and 1:5,000, respectively, in buffer (10 mM Tris-HCl, 100 mM KCl, pH 7.2) containing 10 mM EGTA (zero free Ca^2+^) or 10 mM Ca-EGTA (39 μM free Ca^2+^). These two stocks were mixed in various ratios to give 11 solutions with different free Ca^2+^ concentrations, as described previously^13^. The calculated *Kd* represents the concentration of Ca^2+^ when the fluorescence change of the indicator is half of its maximum value.

Titration of NTnC with 0–500 mM free magnesium was performed according to the observed green fluorescence, as described previously^21^.

In addition, pH titration, photobleaching experiments, and protein maturation are described in the Supplementary Methods.

Size-exclusion chromatography was performed with a Superdex^TM^ 200 10/300 GL column using GE ÄKTA Explorer (Amersham Pharmacia, UK) FPLC System. The concentrations of purified NTnC and GCaMP6s proteins were 20–40 and 10 mg/ml, respectively.

### Stopped-flow fluorimetry

Ca^2+^-binding kinetics experiments were performed on a Chirascan Spectrofluorimeter (Applied Photophysics, UK) equipped with a stopped-flow module at 20 °C. Fluorescence excitation was set to 490 nm, and fluorescence emission was collected using a 515 nm cut-off filter. Three replicates were averaged for analysis. Kinetic records were fitted to either a single or a double exponential using DataFit9 (Oakdale Engineering, USA).

To measure association kinetics, NTnC or GCaMP6s solution (20 μg/ml) in 30 mM HEPES buffer (pH 7.2) containing 100 mM KCl and 1 mM EGTA was rapidly mixed (1:1) with 30 mM HEPES buffer (pH 7.2) containing 100 mM KCl, 10 mM EGTA and increasing Ca^2+^ concentrations. Exponential fitting of the fluorescence signal changes over time provided the relaxation rate constants (k_obs_). Fitting the observed data to the equation k_obs_ = k_on_ × [Ca^2+^]^n^ + k_off_ provided the association rate constant (k_on_) and Hill coefficient (n). K_d kinetic_ = (k_off_/k_on_)^1/n^.

To measure dissociation kinetics, protein solution (20 μg/ml) in 30 mM HEPES (pH 7.2), 100 mM KCl and 1 μM CaCl_2_ was rapidly mixed (1:1) with 30 mM HEPES (pH 7.2), 100 mM KCl, and 10 mM EGTA. Exponential fitting of the fluorescence signal changes over time provided dissociation rate constants (k_off_).

### Cell culture and transfection

HeLa Kyoto or HEK293T cells were maintained in Dulbecco’s Modified Eagle Medium (DMEM) (GIBCO) supplemented with 10% fetal bovine serum (FBS) (Sigma), 2 mM GlutaMax-I (GIBCO), 50 U/ml penicillin, and 50 μg/ml streptomycin (GIBCO). Plasmids for transfection were prepared using a Plasmid Miniprep purification kit (Evrogen, Russia). Transfection was performed using TurboFectTM (Thermo Fisher Scientific, USA) according to the manufacturer’s protocol.

### rAAV particle production and isolation

The rAAV particles were purified as described previously^22^, with some modifications (Supplementary Methods).

### Mammalian live-cell imaging

HeLa Kyoto or HEK293T cell cultures were imaged 24–48 h after transfection using a laser spinning-disk Andor XDi Technology Revolution multi-point confocal system (Andor, UK) equipped with an inverted Nikon Eclipse Ti microscope, a 75 W mercury-xenon lamp (Hamamatsu, Japan), a 60× oil immersion objective (Nikon, Japan), a 16-bit QuantEM 512SC electron-multiplying CCD (Photometrics, USA), and a cage incubator (Okolab, Italy). Before imaging, the culture medium was changed to Dulbecco’s Phosphate Buffered Saline (DPBS) buffered with 20 mM HEPES, pH 7.4.

For time-lapse imaging experiments with varying Ca^2+^ concentration, 1 mM EDTA and 5 μM ionomycin were added to cells for imaging calcium sensors in the Ca^2+^-free state. After imaging calcium indicators in the apo-state, cells were washed with DPBS buffered with 20 mM HEPES, pH 7.4. Next, 2 mM CaCl_2_ and 5 μM ionomycin were added to induce fluorescence signal for Ca^2+^-saturated calcium indicators.

### Isolation, transduction, and imaging of neuronal cultures

Dissociated neuronal cultures were isolated from C57BL/6 mice at postnatal days 0–3 and were grown on 35-mm MatTek glass-bottom dishes in Neurobasal Medium A (GIBCO, UK) supplemented with 2% B27 Supplement (GIBCO, UK), 0.5 mM glutamine (GIBCO, UK), 50 U/ml penicillin, and 50 μg/ml streptomycin (GIBCO, UK). On the 4^th^ day *in vitro*, neuronal cells were transduced with 1–2 μl rAAV viral particles carrying AAV-*CAG*-NTnC, AAV-*CAG*-R-GECO1, AAV-*CAG*-GCaMP6s, or AAV-*CAG*-NTnC/166D+/202D+. Cells were imaged using an Andor XDi Technology Revolution multi-point confocal system.

### Culture and electrophysiology of neuronal cultures plated on MEAs

Neuronal cultures were isolated from new-born C57BL/6 mouse pups (P0) and were plated on 60-channel MEAs (MCS, Germany) (Supplementary Methods). On the 6^th^ day *in vitro* (DIV), 8 cultures (four NTnC and four GCaMP6s) were transduced with rAAV particles carrying NTnC or GCaMP6s sensors under control of the CAG promoter. As a control, 3 cultures were used with no transduction. Electrical activities were registered using a multielectrode registration system *in vitro* MEA60 System (MCS GmbH, Germany). Statistical data were analyzed using the Kolmogorov-Smirnov test (Supplementary Methods).

### Electrophysiology coupled with calcium ion imaging

Whole-cell recordings with patch electrodes were made from neuronal cultures expressing calcium sensors, selected under visual control using 470/20 BP and 510 LP green fluorescence filter sets, Nomarski optics, and infrared video microscopy. The patch electrodes were filled with a potassium gluconate-based solution (130 mM potassium gluconate, 20 mM KCl, 4 mM Mg-ATP, 0.3 mM Na_2_-GTP, 10 mM sodium phosphocreatine, and 10 mM HEPES at pH 7.3) and had a resistance of 6–8 MΩ. During recording, cells were bathed in room-temperature modified Hank’s solution containing 138 mM NaCl, 1.26 mM CaCl_2_, 0.5 mM MgCl_2_, 0.4 mM MgSO_4_, 5.3 mM KCl, 0.44 mM KH_2_PO_4_, 4.16 mM NaHCO_3_, 0.34 mM Na_2_HPO_4_, 10 mM Glucose, and 10 mM HEPES at pH 7.4. Recordings were made with a MultiClamp 700B (Molecular Devices, USA) amplifier in the bridge mode. After amplification and low-pass filtering at 10 kHz, data were digitized at 20 kHz and fed into a computer using the Digidata 1500 interface and PCLAMP software (Molecular Devices, USA). In some experiments, synaptic transmission was blocked by adding 25 μM 2R-amino-5-phosphonopentanoate (APV) and 5 μM 6,7-dinitroquinoxaline-2,3-dione (DNQX) to the extracellular solution. Chemicals were obtained from Sigma-Aldrich or Tocris (USA). Cells were stimulated briefly (5 ms) with intracellularly applied current pulses; the intensity of the pulses was adjusted to reliably induce APs for each cell.

Optical imaging was performed on an Olympus BX51WI microscope equipped with a UV-compatible 40× objective, two camera ports, and a collimated light-emitting diode (LED) with an output power of 650 mW and a peak emission wavelength of 470 nm (Thorlabs, USA) for epi-illumination. Imaging was performed with a NeuroCCD camera (80 × 80 pixels, RedShirt Imaging, USA) using a frame rate of 40 Hz. Fluorescence changes were measured with single-wavelength excitation (470 ± 20 nm) and emission >510 nm. Analysis of optical data, including spatial averaging and high- and low-pass filtering, was conducted with Neuroplex 7 software (RedShirt Imaging, USA). The time-courses of the responses were corrected for bleaching using a linear regression computed through the mean values 2 seconds before the stimulation and by subtracting the extrapolated values.

### Surgery for V1 *in vivo* two-photon imaging

*In vivo* imaging was performed on C57BL/6 (Jackson Laboratory, USA) mice infected with rAAV particles. For cranial window surgery, mice were anesthetized using zoletil (40 mg/kg) and xylazine (5 mg/kg). Next, circular surgery above V1 (centered 2.6 mM right and 3.5 mM posterior to the bregma) was performed in accordance with a previously described protocol^23^. The rAAV particles were injected into the cortex directly after the craniotomy was made. Approximately 0.3 μl of rAAV particles was injected through a glass micropipette into layer 2/3 of the primary visual cortex and into the center of the craniotomy. Then, a 5-mm round glass coverslip (no. 1 thickness) was cemented to the brain using white dental cement (Stoelting, USA). A Neurotar (Neurotar, Ltd., Finland) head post was cemented to the skull and was later used for fixation under the microscope.

### Two-photon *in vivo* mouse imaging in V1

Two-photon imaging of mouse brain was performed 60–65 days after viral transduction using an Olympus MPE1000 two-photon microscope equipped with a Mai Tai Ti:Sapphire femtosecond-pulse laser (Spectra-Physics, USA) and a water-immersion objective lens at 1.05 NA (Olympus, USA). A wavelength of 960 nm was used for excitation. Images were acquired using the Olympus software. Functional images (256 × 256 pixels, 0.429 s per frame) of V1 neurons (460 μm under the pia) were recorded.

As visual stimuli, we used moving gratings generated using PsychoPy^24^, which were presented using an LCD monitor (30 × 50 cm) placed 32 cm in front of the left eye of the mouse. Each stimulus trial consisted of a 10-s blank period (uniform gray display at mean) followed by 10 s of a drifting sinusoidal grating (0.05 cycles/degree, 2 Hz temporal frequency, 8 different directions). Each of the 8 different stimuli was repeated 5 times. Analysis of V1 two-photon functional imaging was performed in FIJI^25^ (Supplementary Methods).

### Animals and surgery for imaging with an nVista HD miniature microscope

Six adult male C57BL/6 mice, aged 20 weeks at the start of the experiments, were used for this study. Mice underwent two surgical procedures under zoletil-xylazine anesthesia (40 and 5 mg/kg, respectively). First, a circular 2-mm-diameter craniotomy was made, and 1 μl of rAAV viral particles (carrying AAV-*CAG*-NTnC or AAV-*CAG*-GCaMP6s) was injected through a glass micropipette into layer 2/3 of the primary visual cortex (left hemisphere; stereotaxic coordinates: −3.2 mM anteroposterior, −2.1 mM mediolateral to the bregma). All exposed surfaces were sealed with KWIK-SIL silicone adhesive (WPI Inc., USA). One week later, the silicone was removed, and the dura matter was extracted from the craniotomy site. Then, a GLP 1040 lens probe (Inscopix Inc., USA) was lowered slowly to a depth of 150 μm while constantly washing the craniotomy site with sterile cortex buffer. Next, all exposed tissue was sealed with KWIK-SIL and white dental cement (Stoelting, USA).

### *Ca* ^*2+*^ *in vivo* imaging with the nVista HD miniature microscope

After a recovery period of at least two weeks, mice were anesthetized again, and base plates for attaching the portable nVista HD miniature microscope (Inscopix Inc., USA) were mounted onto the dental acrylic caps. The two mice (one NTnC and one GCaMP6s) with the best fluorescence characteristics were selected for further imaging. A few days after base plate mounting, we sequentially attached the nVista HD microscope to awake mice that were then placed in a rectangular (24 × 29 cm) cage. Two Ca^2+^ activity movies (5 min long) of awake, freely moving mice were captured at a frame rate of 20 Hz. To record mouse behavior, a CMOS camera from a Samsung Galaxy Ace 4 smartphone was used; synchronization was achieved using several pulses of an incandescent lamp.

Additionally, neuronal reactions from different visual stimuli were examined. Mice were anesthetized with a half-dose of zoletil-xylazine mix (20 mg/kg and 2.5 mg/kg, respectively), and the nVista HD microscope was sequentially attached to the mice. Several types of visual stimuli were presented, including 60-W incandescent lamp pulses, blue LED pulses (465 nm peak), and a moving sinusoidal grating presented with an LCD monitor, as described previously^14^. The grating was generated using Psychophysics Toolbox^26^,^27^ in MATLAB. Each presentation consisted of a 6-s blank period (uniform gray monitor), followed by a 6-s drift period (sinusoidal grating, moving in a specified direction; we tested various sets of parameters: 0.5–3 Hz temporal frequency, 0.05–0.5 cycles per degree). In total, 40 presentations for 8 different directions (changing clockwise) were made with the 52 × 32 cm LCD monitor placed 25 cm in front of the right eye of the mouse. Image analysis for the nVista HD miniature microscope was performed using Mosaic software (Inscopix Inc., USA) and custom MATLAB scripts (Supplementary Methods).

### Animal care

All methods for animal care and use were approved by the National Research Center “Kurchatov Institute” Committee on Animal Care (protocol No. 1, 7 September 2015) and were in accordance with the *Russian* Federation Order Requirements N *267* МЗ and the National Institutes of Health Guide for the Care and Use of Laboratory Animals. Twenty-nine C57BL/6 mice were used in this study, ages P0 and ~2–6 months old. Mice were used without regard to gender.

## Additional Information

**How to cite this article**: Barykina, N. V. *et al.* A new design for a green calcium indicator with a smaller size and a reduced number of calcium-binding sites. *Sci. Rep.* **6**, 34447; doi: 10.1038/srep34447 (2016).

## Supplementary Material

Supplementary Information

Supplementary Video 1

Supplementary Video 2

## Figures and Tables

**Table 1:** *In vitro* properties of purified NTnC compared to GCaMP6s.

		Proteins			
		NTnC		GCaMP6s	
Properties		apo	sat	apo	sat
Absorbance maximum (nm)		505		402	500
Emission maximum (nm)		518		518	515
Quantum yield^a^		0.71 ± 0.05	0.65 ± 0.04	0.11 ± 0.01	0.61
ε (mM^−1^ cm^−1^)^b^		108 ± 6	52 ± 1^c^	33.3 ± 0.6	77 ± 3
Brightness (%)		163	72	7.8	100
Fluorescence contrast (fold)	0 mM Mg^2+^	2.0 ± 0.3		44 ± 6	
	1 mM Mg^2+^	2.0 ± 0.7		47 ± 24	
pKa		6.09 ± 0.07	6.08 ± 0.02	9.6 ± 0.3	6.16 ± 0.08
K_d_ (nM)	0 mM Mg^2+^	84 ± 6 [n = 1.9 ± 0.1]^d^		144 ± 9 [n = 4.0 ± 0.6]^d^	
	1 mM Mg^2+^	192 ± 40		227.3 ± 0.2	
K_d_^kin^ (nM)^e^		94 ± 9 [n = 2.3 ± 0.1]		152 ± 20 [n = 3.2 ± 0.1]	
k_on_ (s^−1^ × M^−n^)^e^		6 × 10^15^		6 × 10^21^	
k_off_ (s^−1^)^f^		0.8 ± 0.1; 0.05 ± 0.01^g^		0.8 ± 0.1	
k^onset^_limit_ (s^−1^)^h^		50 ± 20		270 ± 50	
Protein state		monomer		monomer	
Maturation half-time (min)^i^		23	28	ND	ND
Photobleaching half-time (sec)^j^		40 ± 8	70 ± 5	111 ± 15	186 ± 13

^a^GCaMP6s in the saturated state (QY = 0.61 ref. 14) and mTagBFP2 (QY = 0.64 ref. 28) were used as reference standards for 500- to 505- and 402-nm absorbing states, respectively.

^b^Extinction coefficient was determined by alkaline denaturation.

^c^Extinction coefficient was estimated relative to apo NTnC with the same concentration.

^d^Hill coefficient is shown in square brackets.

^e^K_d_^kin^, Hill coefficients and k_on_ values were obtained via fitting the observed association rates (Supplementary Fig. 8a) at 0–350 nM Ca^2+^ concentrations to the equation k_obs_ = k_on_ × [Ca^2+^]^n^ + k_off_ (Fig. 2d). K_d_^kinetic^ = (k_off_/k_on_)^1/n^. Hill coefficients are shown in square brackets.

^f^Refined k_off_ values were determined from the dissociation kinetics records (Supplementary Fig. 8b).

^g^Unlike GCaMP6s kinetics, NTnC kinetics do not agree with the two-state model. NTnC kinetic curves were fitted to double exponentials. k_off_ values were estimated from double exponential decay with individual exponent contributions of 0.48:0.52.

^h^k^onset^_limit_ values are saturation levels of the observed association rates (at >600–800 nM Ca^2+^ concentrations; Supplementary Fig. 9).

^i^mEGFP had a maturation half-time of 14 min; we could not estimate the maturation rate for GCaMP6s because of its low expression level in bacteria.

^j^mEGFP had a photobleaching half-time of 170 ± 20 sec.

**Table 2:** Characteristics of calcium ion responses to intracellular stimulation with 1 and 10 APs in neurons expressing the sensors NTnC and GCaMP6s in dissociated neuronal culture.

Protein	Number of cells	APs	Rise half-time, s^a^	Decay half-time, s^b^	ΔF/F_0_^c^	SNR^d^
NTnC	6	1	0.15 ± 0.02	2.7 ± 0.5	0.027 ± 0.008	18 ± 4
	8	10	0.21 ± 0.01	4.5 ± 0.8	0.15 ± 0.02	80 ± 20
GCaMP6s	5	1	0.19 ± 0.03	2.7 ± 0.3	0.022 ± 0.008	25 ± 9
	5	10	0.26 ± 0.01	3.6 ± 0.5	0.60 ± 0.20	400 ± 200

^a^Rise half-time was measured as the time between the stimulus onset and the half-peak of response.

^b^Decay half-time was calculated as the time from the peak to the half-peak at the end of the response.

^c^ΔF/F_0_ was calculated as (F − F_0_)/F_0_, where F_0_ is the baseline fluorescence signal averaged over a 1-s period immediately after the start of imaging.

^d^Signal-to-noise ratio (SNR) was quantified as the peak ΔF/F_0_ response over the standard deviation of the signal during a one-second period before stimulation. Values are shown as the means ± standard errors of the mean.
