# Supplementary material for: A new design for a green calcium indicator with a smaller size and a reduced number of calcium-binding sites
Source: Sci Rep. 2016 Sep 28;6:34447. doi: 10.1038/srep34447 (PMC5039633; doi:10.1038/srep34447)
Supplement: Supplementary Information [file srep34447-s1.pdf]

A new design for a green calcium indicator with a smaller size and a reduced number of calcium-binding sites.

Natalia V. Barykina,[1,2] Oksana M. Subach,[1,3] Danila A. Doronin,[1,3] Vladimir P. Sotskov,[1] Marina A. Roshchina,[3] Tatiana A. Kunitsyna,[3] Aleksey Y. Malyshev,[4] Ivan V. Smirnov,[4,5] Asya M. Azieva,[3] Ilya S. Sokolov,[3] Kiryl D. Piatkevich,[6] Mikhail S. Burtsev,[3] Anna M. Varizhuk,[7,8] Galina E. Pozmogova,[7] Konstantin V. Anokhin,[1,2,3] Fedor V. Subach[1*] and Grigori N. Enikolopov[1,9,10,11*]

[1] NBICS Department, Moscow Institute of Physics and Technology, Moscow 123182, Russia
[2] Department of Systems Neurobiology and Functional Neurochemistry, P.K. Anokhin Institute of Normal Physiology of RAMS, Moscow, 125009, Russia
[3] Department of Neuroscience, National Research Center "Kurchatov Institute", Moscow, 123182, Russia
[4] Laboratory of Cellular Neurobiology of Learning, Institute of Higher Nervous Activity and Neurophysiology of RAS, Moscow, 117485, Russia
[5] Medico-Biological Faculty, N. I. Pirogov Russian National Research Medical University, Moscow, Russia
[6] MIT Media Lab, Massachusetts Institute of Technology, Cambridge, MA 02139, USA
[7] Federal Research and Clinical Center of Physical-Chemical Medicine of Federal Medical Biological Agency, 119435, Moscow, Russia
[8] Engelhardt Institute of Molecular Biology RAS, 119991, Moscow, Russia
[9] Cold Spring Harbor Laboratory, Cold Spring Harbor, NY 11724, USA
[10] Department of Anesthesiology, Stony Brook University Medical Center, NY 11794, USA
[11] Center for Developmental Genetics, Stony Brook University, NY 11794, USA

*Correspondence and requests for materials should be addressed to F.V.S. (email: subach.fv@mipt.ru and subach_fv@rrcki.ru) or G.N.E. (email: Grigori.Enikolopov@stonybrookmedicine.edu)

Supplementary Information

Supplementary Figures, Tables and Videos

Supplementary Figure 1. Alignment of the amino acid sequences for original library and NTnC calcium sensor.

Supplementary Figure 2. Alignment of the amino acid sequences of mNeonGreen with EGFP, EYFP and TurboGFP.

Supplementary Figure 3. A schematic representation of NTnC libraries design with variable linkers.

Supplementary Figure 4. The pH dependence of absorbance for NTnC indicator.

Supplementary Figure 5. The pH dependence of fluorescence and dynamic range for GCaMP6s.

Supplementary Figure 6. Calcium titration and pH dependence of fluorescence for mutant NTnC/166D+/202D+ with eliminated calcium activity.

Supplementary Figure 7. Magnesium titration of NTnC sensor.

Supplementary Figure 8. Kinetics curves for stopped-flow experiments.

Supplementary Figure 9. Observed association rates at high Ca^{2+} concentrations (onset rate limits) acquired by stopped-flow experiments.

Supplementary Figure 10. Size-exclusion chromatography for NTnC and control GCaMP6s.

Supplementary Figure 11. Response of NTnC sensor to variations of Ca^{2+} concentration in HeLa cells and neuronal culture.

Supplementary Figure 12. Example of non-averaged fluorescent response of NTnC expressing neuron to train of action potentials induced by intracellular injection of depolarizing current step.

Supplementary Figure 13. Ca^{2+} titration curves for NTnC and GCaMP6s in the absence or in the presence of 1 mM $MgCl_2$.

Supplementary Figure 14. Time dependence of spiking rate for neuronal cultures plated on MEAs.

Supplementary Figure 15. Characterization of interburst intervals for neuronal cultures plated on MEAs.

Supplementary Figure 16. Characterization of a number of spikes in burst for neuronal cultures plated on MEAs.

Supplementary Figure 17. Reaction of NTnC and GCaMP6s calcium indicators on simple visual stimuli *in vivo* registered with nVista HD.

Supplementary Figure 18. Reaction of NTnC and GCaMP6s calcium indicators on complex visual stimuli *in vivo* registered with nVista HD.

Supplementary Table 1. Characteristics of calcium responses for neurons expressing NTnC and GCaMP6s sensors during spontaneous activity of neuronal dissociated cultures.

Supplementary Table 2. Electrophysiological characteristics for spontaneous activity of neuronal cultures plated on MEAs.

Supplementary Table 3. Characteristics of calcium responses in neurons expressing NTnC and GCaMP6s sensors in brain cortex of freely moving mice registered with nVista HD.

Supplementary Table 4. Characterization of *in vivo* calcium activities for neurons expressing NTnC and GCaMP6s sensors in brain cortex of anesthetized mice during presentation of simple visual stimuli registered with nVista HD.

Supplementary Table 5. Characterization of *in vivo* calcium activities for neurons expressing NTnC and GCaMP6s sensors in brain cortex of anesthetized mice during presentation of complex visual stimuli registered with nVistanVista HD.

Supplementary Table 6. List of primers.

Supplementary Video 1. ***In vivo* neuronal calcium activity in freely behaving mice visualized with the NTnC calcium indicator and a nVista HD system.** A video of neuronal calcium activity in visual cortex of mouse displayed as relative changes in fluorescence ($\Delta F/F$) at 20 Hz frame rate **(left panel)** and the synchronously acquired movie showing a mouse exploring a cage **(right panel)**.

Supplementary Video 2. ***In vivo* neuronal calcium activity in freely behaving mice visualized with the GCaMP6s calcium indicator and a nVista HD system.** A video of neuronal calcium activity in visual cortex of mouse displayed as relative changes in fluorescence ($\Delta F/F$) at 20 Hz frame rate **(left panel)** and the synchronously acquired movie showing a mouse exploring a cage **(right panel)**.

Supplementary Methods.

Supplementary Results.

```
                  10        20        30        40        50        60
                   |         |         |         |         |         |
Library   MVSKGEEDNMASLPATHELHIFGSINGVDFDMVGQGTGNPNDGYEELNLKSTKGDLQFSP
NTnC      MVSKGEEDNMASLPATHELHIFGSINGVDFDMVGQGSGNPNVGYEELNLKSTKGDLQFSP

                  70        80        90       100       110       120
                   |         |         |         |         |         |
Library   WILVPHIGYGFHQYLPYPDGMSPFQAAMVDGSGYQVHRTMQFEDGASLTVNYRYTYEGSH
NTnC      WILVPHIGYGFHQYLPYPDGMSPFQAAMVDGSGYQVHRTVQFEDGASLTVNYRYTYEGSH

                 130       140       150       160       170       180
                   |         |         |         |         |         |
Library   IKGEAQVKGTGFPADGPVMTNSLTAXXXSEEELSECFRIFDKDGNGFIDREEFGDIIRLT
NTnC      IKGEAQVKGTGFPADGPVMANSLTAMVPSEEELSECFRTFDKDGDGFIDREEFGGIIRLT
                 190       200       210       220       230       240
                   |         |         |         |         |         |
Library   GEQLTDEDPDEIFGDSDTDKNGRIDFDEFLKMVENVQXXXADWCRSKKTYPNDKTIISTF
NTnC      GEQLTDEDPDEIFGDSDTDKNGRIDFDEFLKMVENVQLSMADWCRSKMACPNDKTLISTL

                 250       260       270       280       290       300
                   |         |         |         |         |         |
Library   KWSYTTGNGKRYRSTARTTYTFAKPMAANYLKNQPMYVFRKTELKHSKTELNFKEWQKAF
NTnC      KWSYTTGNGKRYRSTARTTYTFAKPMAANYLKNQPMYVFRKTELKHSKTELNFKEWQKAF

                 310
                   |
Library   TDVMGMDELYK
NTnC      TDVMGMDELYK
```

Supplementary Figure 1. Alignment of the amino acid sequences for original library and NTnC calcium sensor. Alignment numbering follows that of NTnC. Residues from fluorescent part buried in β-can are highlighted with green. Residues that are forming chromophore are underlined. TnC part is highlighted with yellow color. Mutations in NTnC related to the initial library including two 3 amino acid-linkers are highlighted by red color. E3-E4 calcium binding domains are highlighted with blue color.

```
                        10        20        30        40        50
                         |         |         |         |         |
GFP             MSKGEELFTGVVPILVELDGDVNGHKFSVSGEGEGDATYGKLTLKFICTTG---KLPVPWP
EYFP           MVSKGEELFTGVVPILVELDGDVNGHKFSVSGEGEGDATYGKLTLKFICTTG---KLPVPWP
TurboGFP         MESDESGLPAMEIECRITGTLNGVEFELVGGGEGTPEQGRMTNKMKSTKG---ALTFSPY
LanYFP                 MSLPATHELHIFGSFNGVDFDMVGRGTGNPNDGYEELNLKSTKG---ALQFSPW
mNeonGreen     MVSKGEEDNMASLPATHELHIFGSINGVDFDMVGQGTGNPNDGYEELNLKSTKG---DLQFSPW

                60        70        80        90       100       110
                 |    ***   |         |         |         |         |
GFP             TLVTTFSYGVQCFSRYPDHMKQHDFFKSAMPEGYVQERTIFFKDDGNYKTRAEVKFEGDT
EYFP            TLVTTFGYGLQCFARYPDHMKQHDFFKSAMPEGYVQERTIFFKDDGNYKTRAEVKFEGDT
TurboGFP        LLSHVMGYGFYHFGTYPSGYE-NPFLHAINNGGYTNTRIEKYEDGGVLHVSFSYRYEAGR
LanYFP          ILVPQIGYGFHQYLPFPDGMS-PFQAAMKDGSGYQVHRTMQFEDGASLTSNYRYTYEGSH
mNeonGreen      ILVPHIGYGFHQYLPYPDGMS-PFQAAMVDGSGYQVHRTMQFEDGASLTVNYRYTYEGSH

               120       130       140       150       160       170
                 |         |         |         |         |         |
GFP             LVNRIELKGIDFKEDGNILGHKLEYNYNSHNVYIMADKQKNGIKVNFKIRHNIEDGSVQL
EYFP            LVNRIELKGIDFKEDGNILGHKLEYNYNSHNVYIMADKQKNGIKVNFKIRHNIEDGSVQL
TurboGFP        VIGDFKVMGTGFPEDSVIFTDKII-RSNATVEHLHPM-GDNDLDGSFTRTFSLRDGGYYS
LanYFP          IKGEFQVIGTGFPADGPVMTNSLT-AADWCVTKMLYP-NDKTIISTFDWTYTTGSGKRYQ
mNeonGreen      IKGEAQVKGTGFPADGPVMTNSLT-AADWCRSKKTYP-NDKTIISTFKWSYTTGNGKRYR
                                          ↑

             180       190          200       210       220       230
               |         |            |         |         |         |
GFP            ADHYQQNTPIGDG---PVLLPDNHYLSTQSALSKDPNEKRDHMVLLEFVTAAGITHGMDELYK
EYFP           ADHYQQNTPIGDG---PVLLPDNHYLSYQSALSKDPNEKRDHMVLLEFVTAAGITLGMDELYK
TurboGFP       SVVDSHMHFKSAIHPSILQNGGPMFAFRRVEEDHS----NTELGIVEYQHAFKTPDADAGEE
LanYFP         STVRTNYTFAKPMAANILKNQP-MFVFRKTELKHS----KTELNFKEWQKAFTDVM
mNeonGreen     STARTTYTFAKPMAANYLKNQP-MYVFRKTELKHS----KTELNFKEWQKAFTDVMGMDELYK
```

Supplementary Figure 2. Alignment of the amino acid sequences of mNeonGreen with EGFP, EYFP and TurboGFP. Alignment numbering follows that of avGFP. Residues buried in β-can are shaded. Stars indicate residues that are forming chromophore. F165 of GFP corresponding to F240L mutation in NTnC (Supplementary Figure 1) is highlighted by yellow color. Site of insertion in mNeonGreen and EYFP is indicated with arrow.

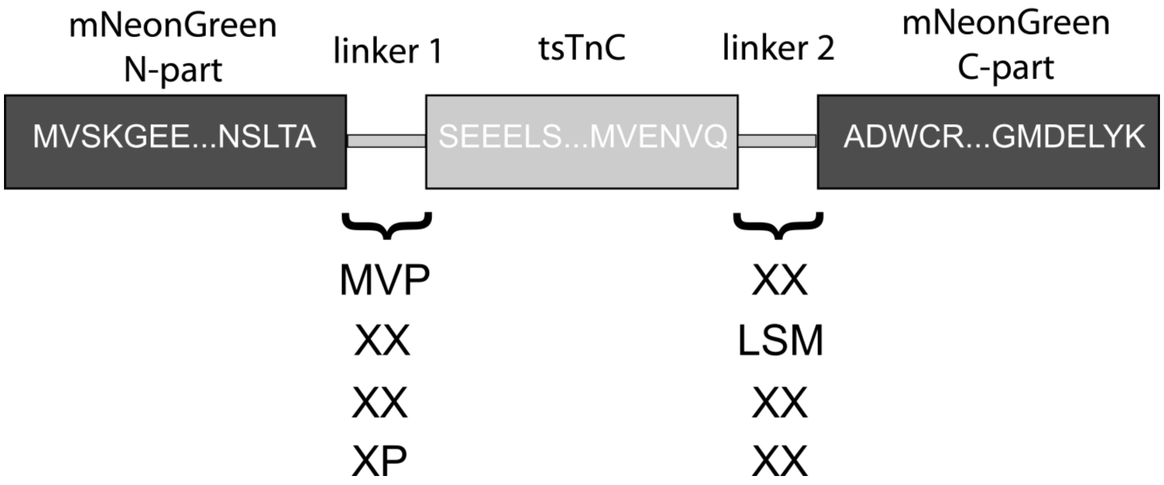

Supplementary Figure 3. A schematic representation of NTnC libraries design with variable linkers. The minimal calcium-binding domain tsTnC (grey) is sandwiched via amino acid linkers between N- and C- parts of mNeonGreen fluorescent protein (dark grey). Linkers used in final version of sensor are MVP and LSM.

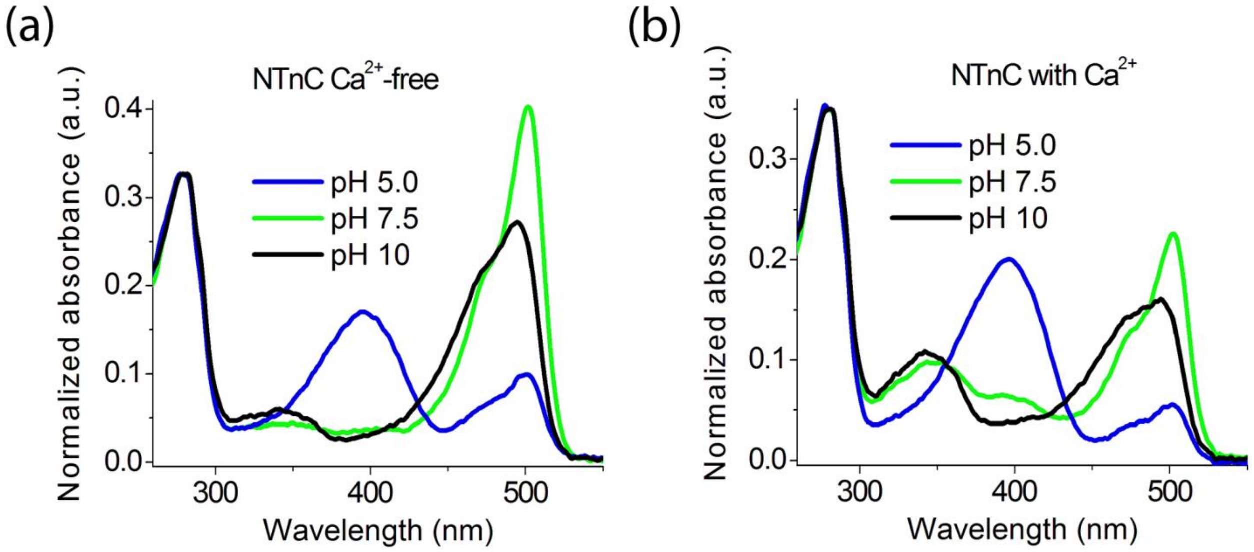

Supplementary Figure 4. The pH dependence of absorbance for NTnC indicator. Spectra were measured in the presence of 3mM EDTA (**a**) or 3mM $CaCl_2$ (**b**) in 30mM citric-borate, 30mM NaCl buffer.

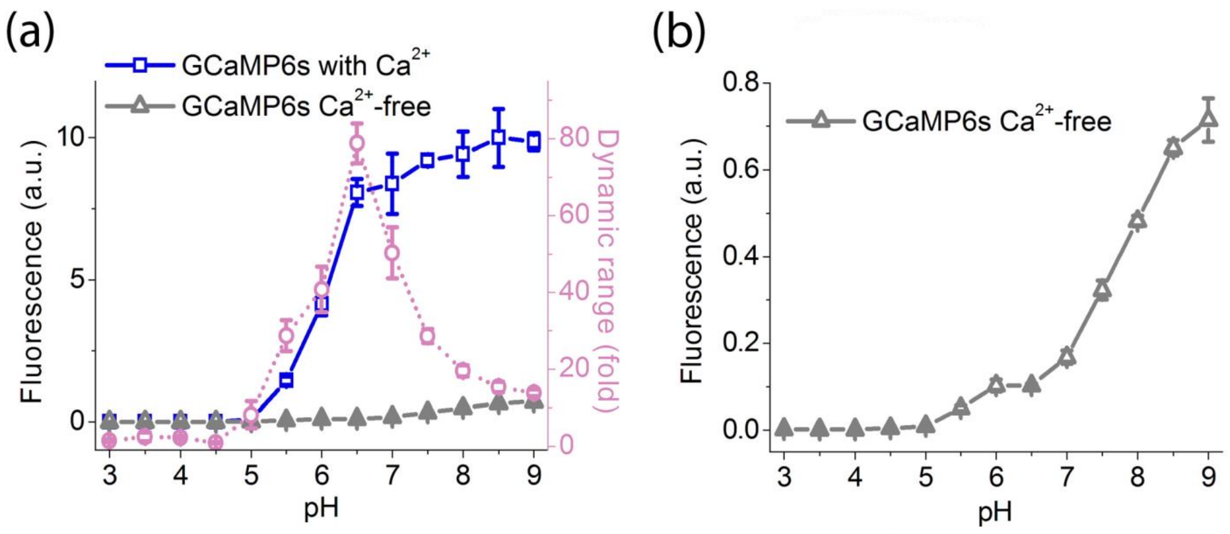

Supplementary Figure 5. The pH dependence of fluorescence and dynamic range for GCaMP6s. (a) Intensity and dynamic range of GCaMP6s as a function of pH. The dynamic range (fold) at each pH value was calculated as the ratio of GCaMP6s fluorescence intensity in the presence of Ca^{2+} to that in the absence of Ca^{2+}. Experimental pKa values are summarized in the Table 1. **(b)** Expanded intensity of GCaMP6s in Ca^{2+}-free state shown in (**a**) as a function of pH.

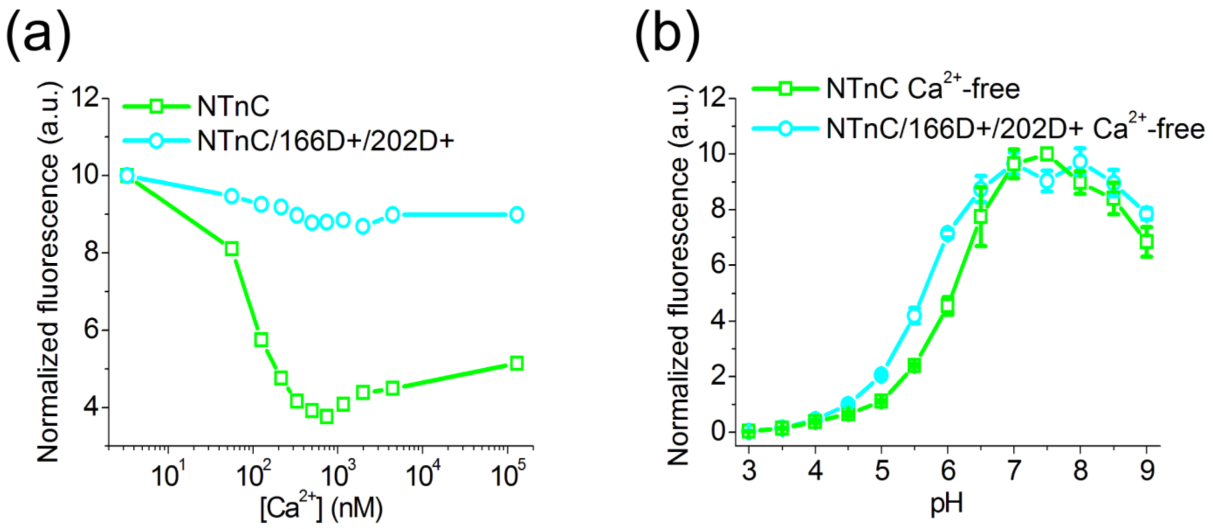

Supplementary Figure 6. Calcium titration and pH dependence of fluorescence for mutant NTnC/166D+/202D+ with eliminated calcium activity. (a) Ca^{2+} titration curves for NTnC and NTnC/166D+/202D+ mutant. **(b)** pH dependence of green fluorescence for NTnC and NTnC/166D+/202D+ mutant (both in Ca^{2+}-free state).

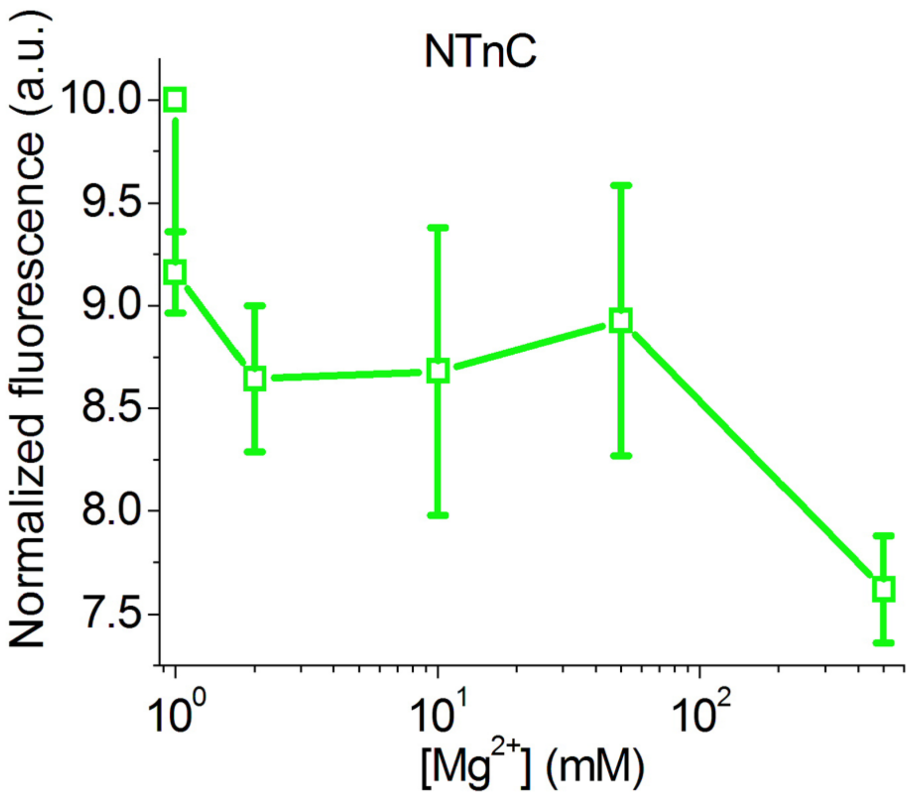

Supplementary Figure 7. Magnesium titration of NTnC sensor.

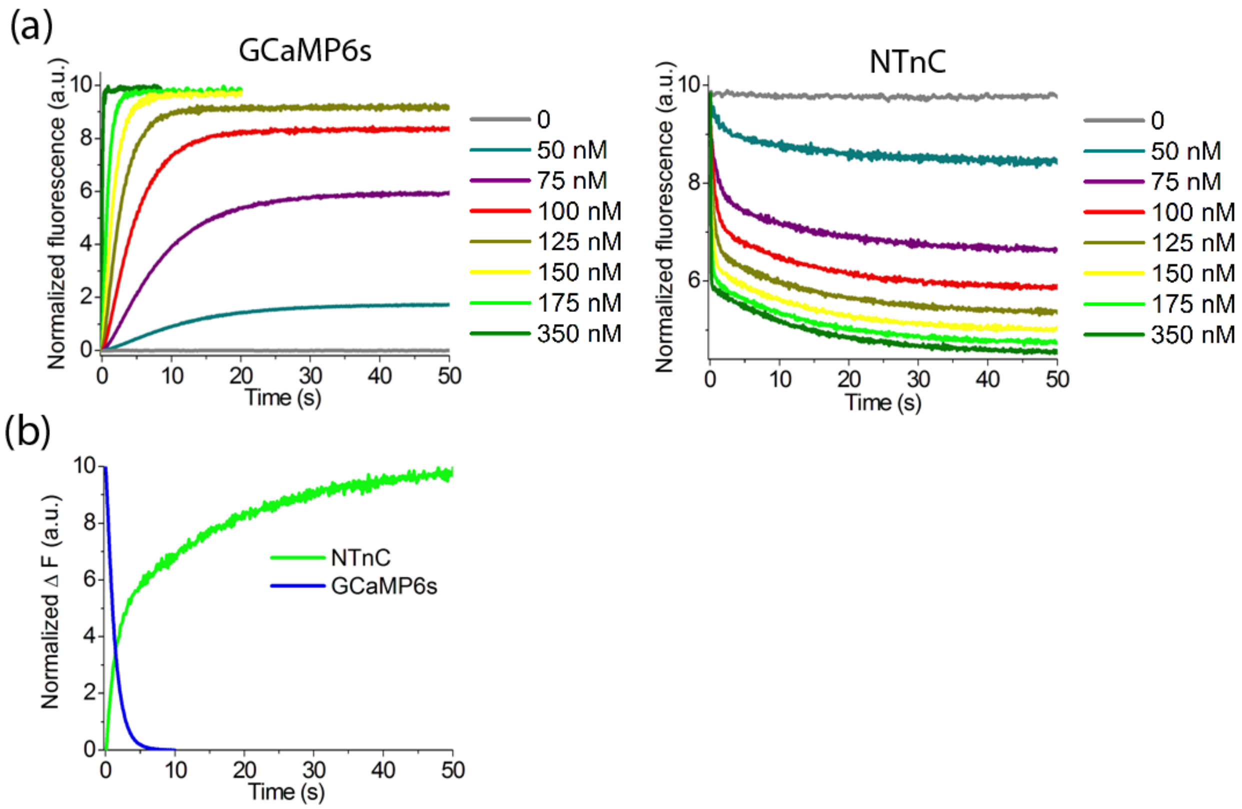

Supplementary Figure 8. Kinetics curves for stopped-flow experiments. (a) Association kinetics. Ca^{2+} concentrations in the mixing chamber are specified in the figure legends. **(b)** Dissociation kinetics. Fluorescence changes are normalized to F_0 of 0 and F_{max} of 10 for NTnC and vice versa for GCaMP6s. All experiments were performed in triplicate and averaged.

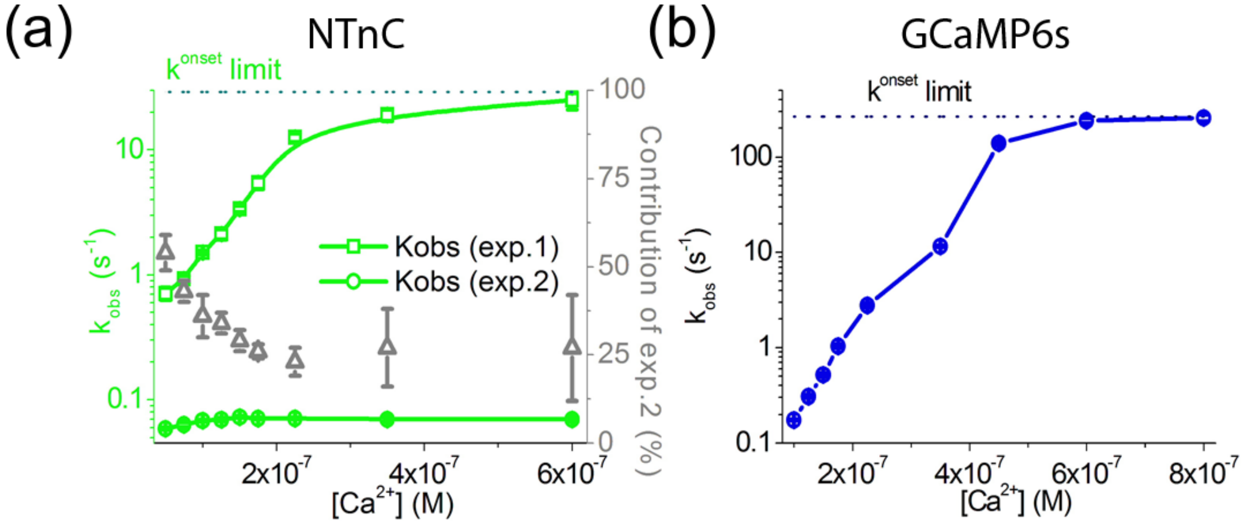

Supplementary Figure 9. Observed association rates at high Ca^{2+} concentrations (onset rate limits) acquired by stopped-flow experiments. k^{onset} limit values corresponding to saturation levels of the observed association rates (at >600-800 nM Ca^{2+} concentrations) of NTnC **(a)** and GCamP6s **(b)** are shown. NTnC kinetic curves were fitted to double exponentials and observed association rates for these two exponential fits as well as contribution of second exponent are shown.

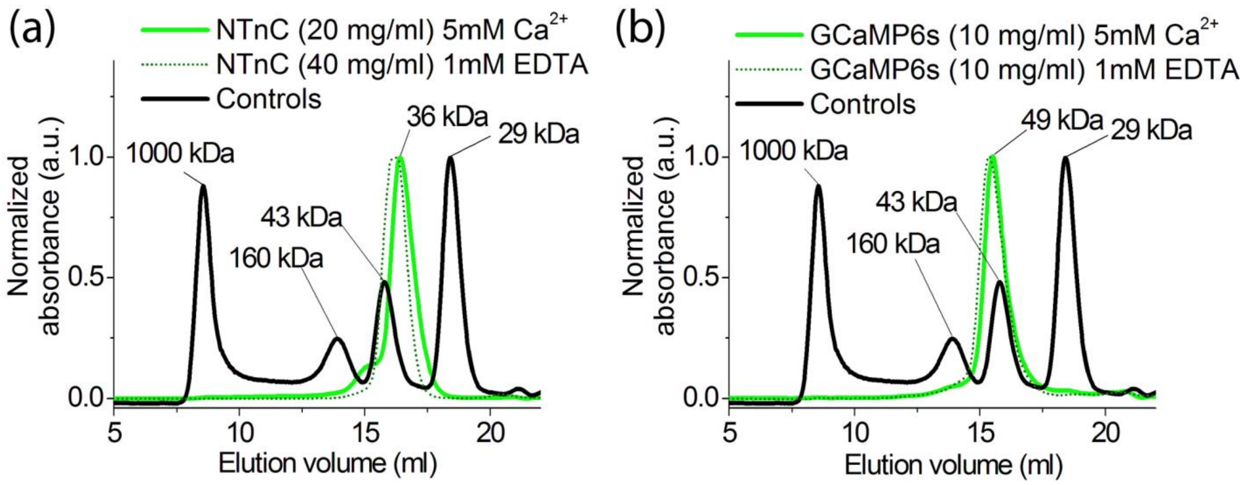

Supplementary Figure 10. Size-exclusion chromatography for NTnC and control GCaMP6s. Fast protein liquid chromatography of NTnC **(a)** and GCaMP6s **(b)** in 40mM Tris-HCl (pH7.5), 200mM NaCl, 5% glycerol buffer supplemented with 5 mM $CaCl_2$ or 1mM EDTA.

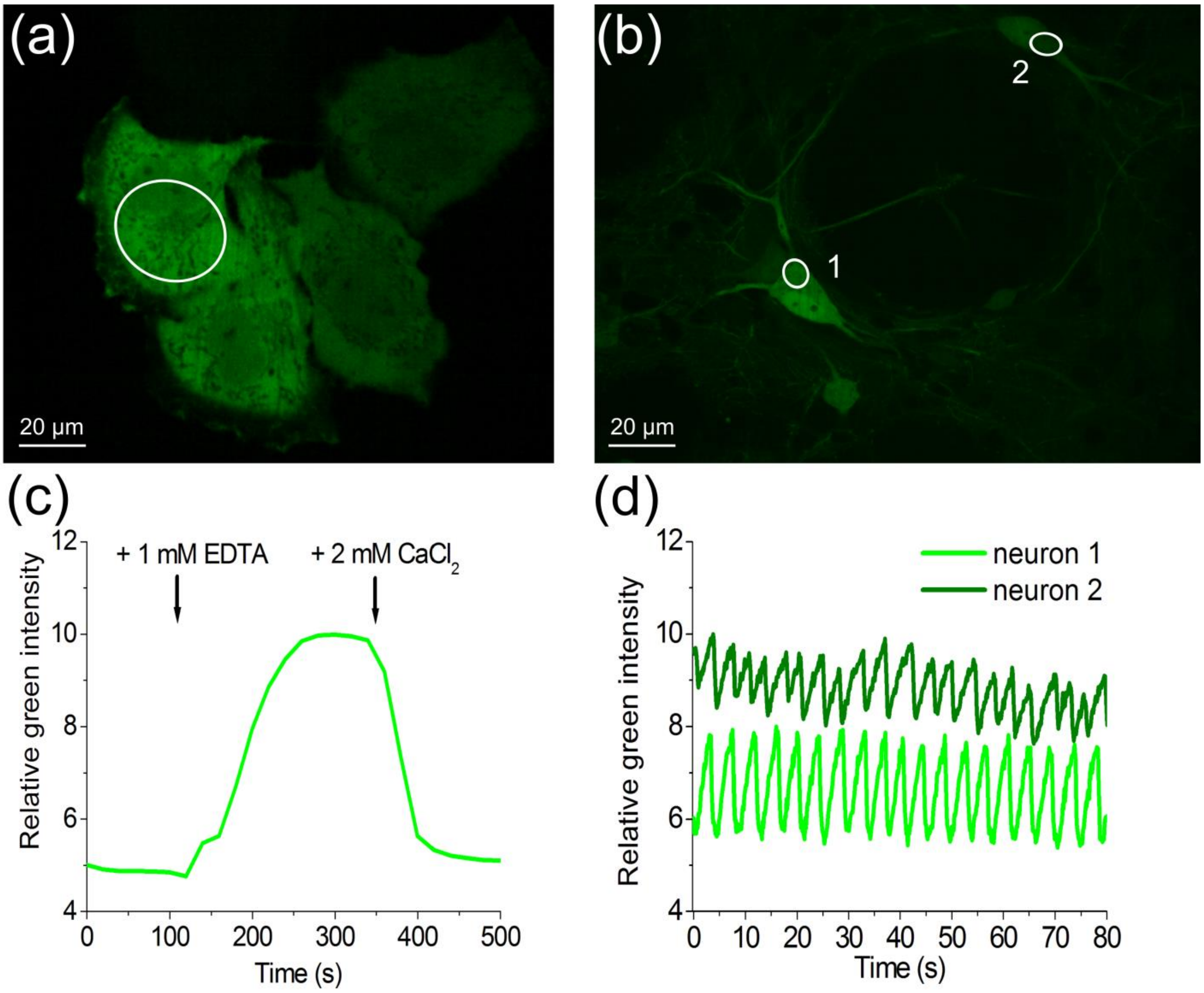

Supplementary Figure 11. Response of NTnC sensor to variations of Ca^{2+} concentration in HeLa cells and neuronal culture. (a) NTnC expressed in HeLa Kyoto cell line transfected with pAAV-*CAG*-NTnC plasmid. **(b)** Dissociated cortical neuronal culture transduced with AAV-CAG-NTnC varal particles. **(c)** The graph illustrates green fluorescence changes in response to addition of 1 mM EDTA together with 5μM Ionomycin and 2 mM $CaCl_2$ mixed with 5μM Ionomycin. **(d)** NTnC green fluorescence changes were captured during spontaneous neuronal activity. (**c**), (**d**) The graphs illustrate green fluorescence changes in respective area selected with white circle.

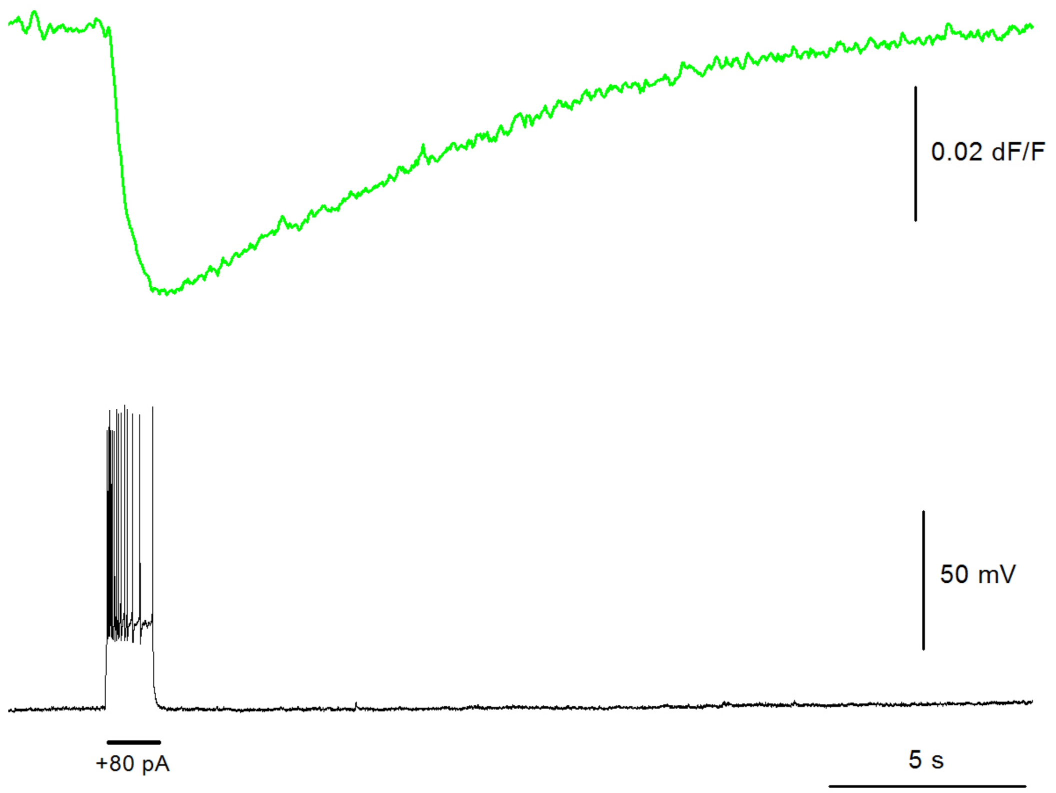

Supplementary Figure 12. Example of non-averaged fluorescent response of NTnC expressing neuron to train of action potentials induced by intracellular injection of depolarizing current step.

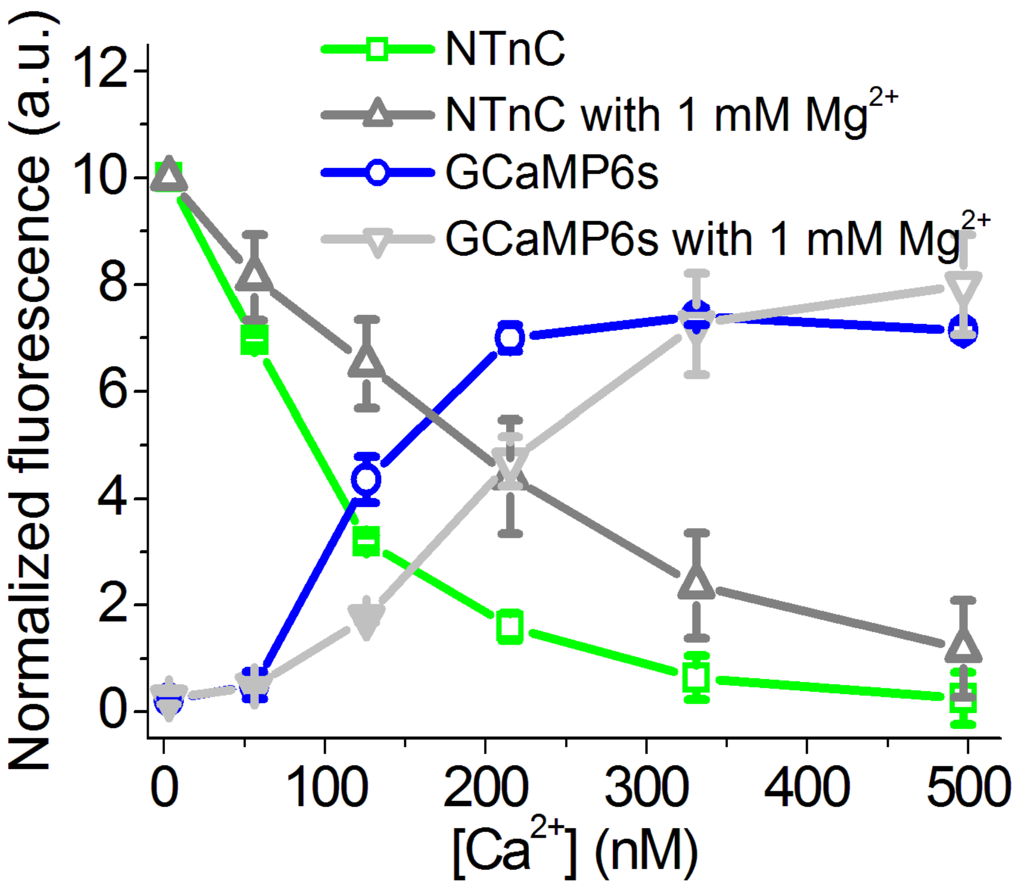

Supplementary Figure 13. Ca^{2+} **titration curves for NTnC and GCaMP6s in the absence or in the presence of 1 mM** $MgCl_2$**.**

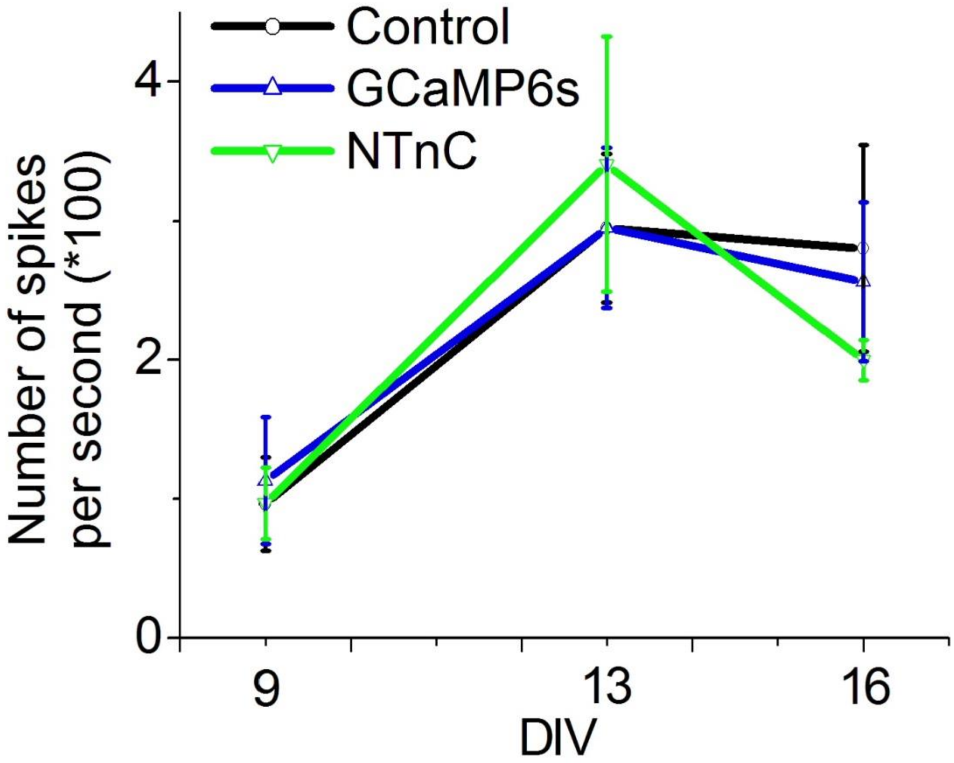

Supplementary Figure 14. Time dependence of spiking rate for neuronal cultures plated on MEAs. Control, GCaMP6s and NTnC correspond to neuronal cultures non-transduced, and transduced with rAAV particles carrying GCaMP6s and NTnC genes, respectively. **(a)**, **(b)**, **(c)** and **(d)** correspond to days in vitro 9, 13, and 16, respectively.

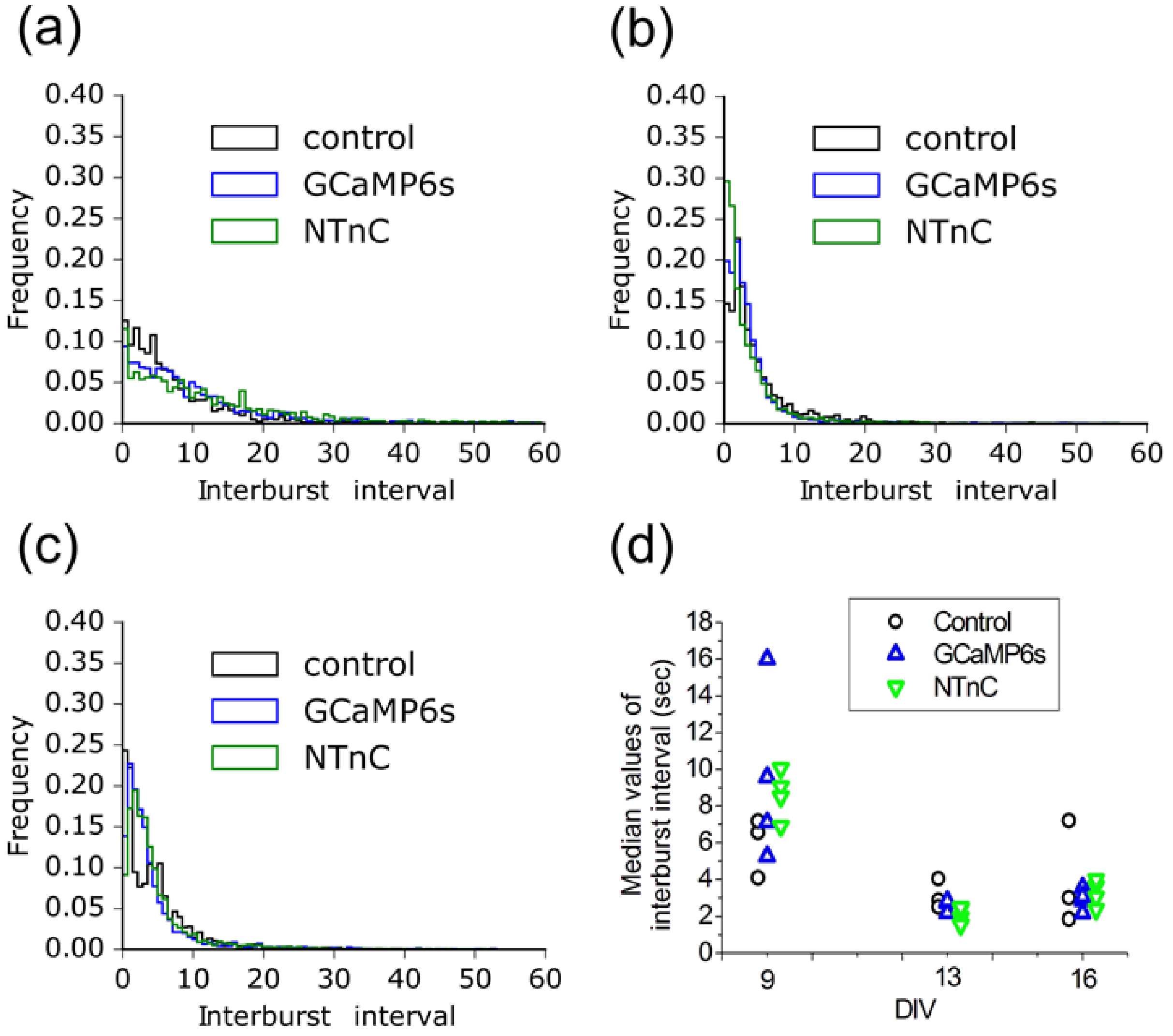

Supplementary Figure 15. Characterization of interburst intervals for neuronal cultures plated on MEAs. Control, GCaMP6s and NTnC correspond to neuronal cultures non-transduced, and transduced with rAAV particles carrying GCaMP6s and NTnC genes, respectively. **(a)**, **(b)**, and **(c)** correspond to summarized distributions for days in vitro 9, 13, and 16, respectively. **(d)** Spreads of median values of interburst interval calculated from individual distributions are shown for all days tested.

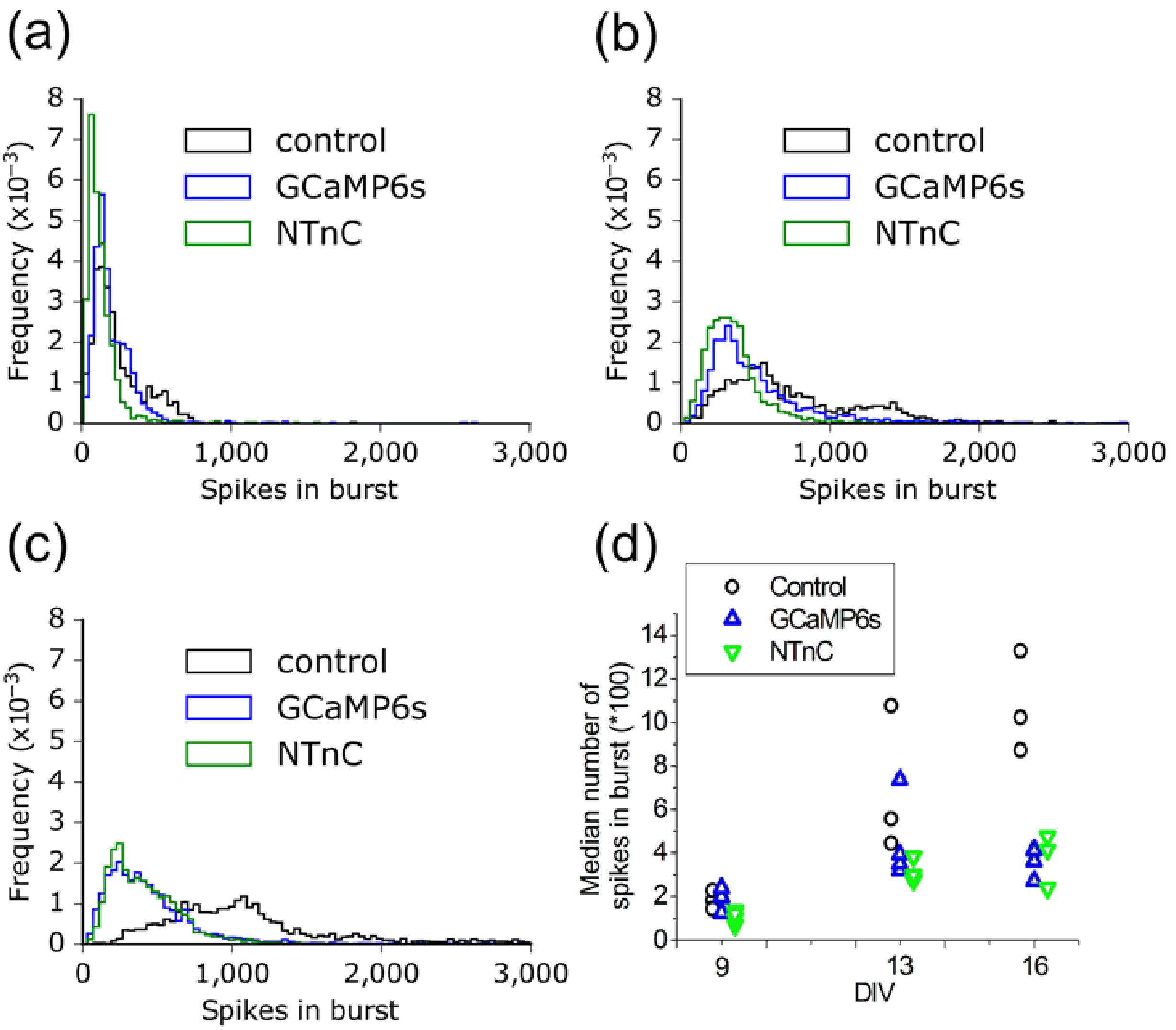

Supplementary Figure 16. Characterization of a number of spikes in burst for neuronal cultures plated on MEAs. Control, GCaMP6s and NTnC correspond to neuronal cultures non-transduced, and transduced with rAAV particles carrying GCaMP6s and NTnC genes, respectively. **(a)**, **(b)**, and **(c)** correspond to summarized distributions for day in vitro 9, 13, and 16, respectively. **(d)** Spreads of median values of a number of spikes in burst calculated from individual distributions are shown for all days tested.

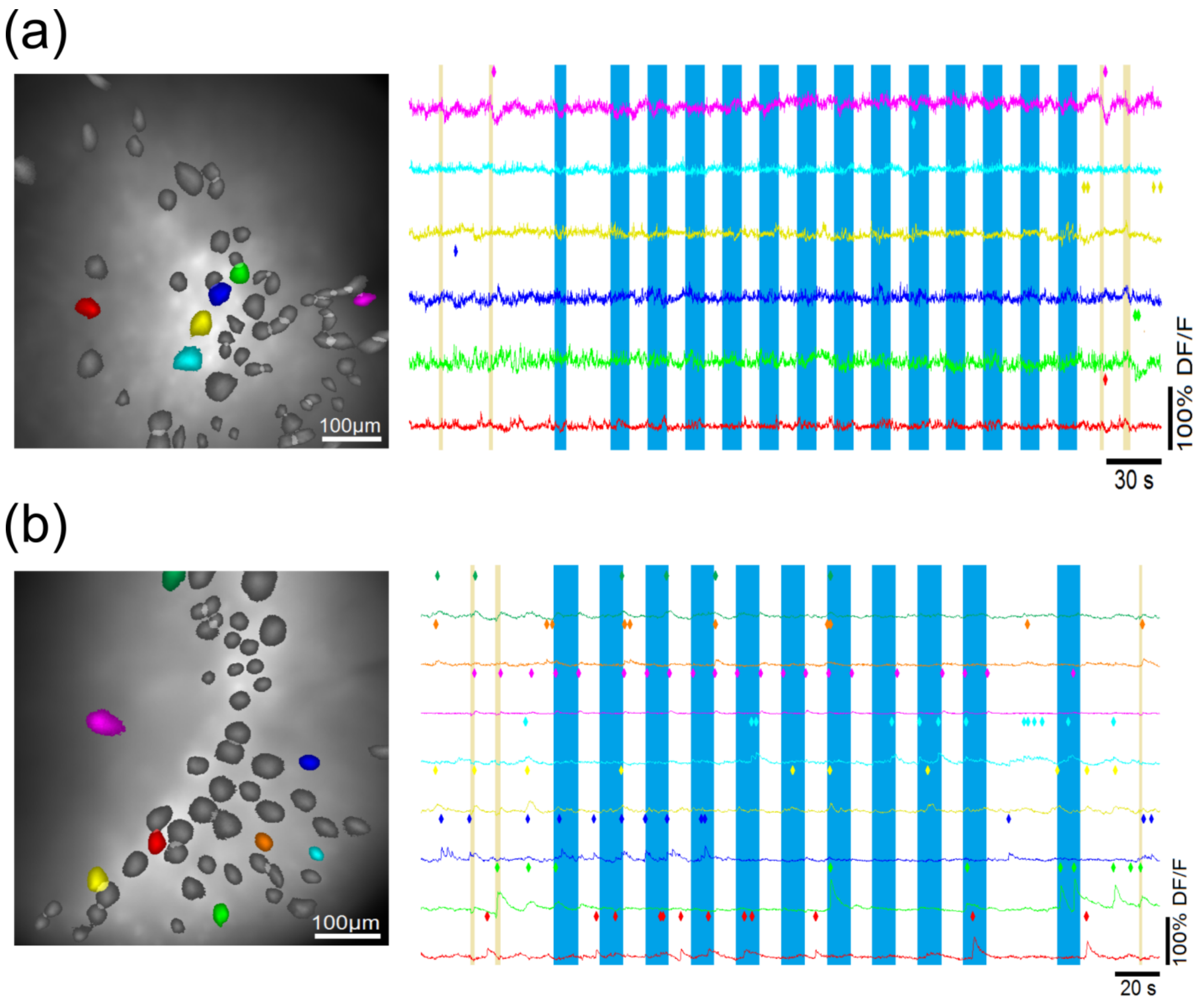

Supplementary Figure 17. Reaction of NTnC and GCaMP6s calcium indicators on simple visual stimuli *in vivo* registered with nVista HD. (a) Spatial filters and sample traces obtained from imaging session with light anesthetized mouse expressing NTnC; visual stimuli consisted of 60W incandescent lamp (yellow strips) and blue 465 nm LED (blue strips) pulses were presented during the session. **(b)** Spatial filters and sample traces obtained from imaging session with mouse expressing GCaMP6s under the same conditions.

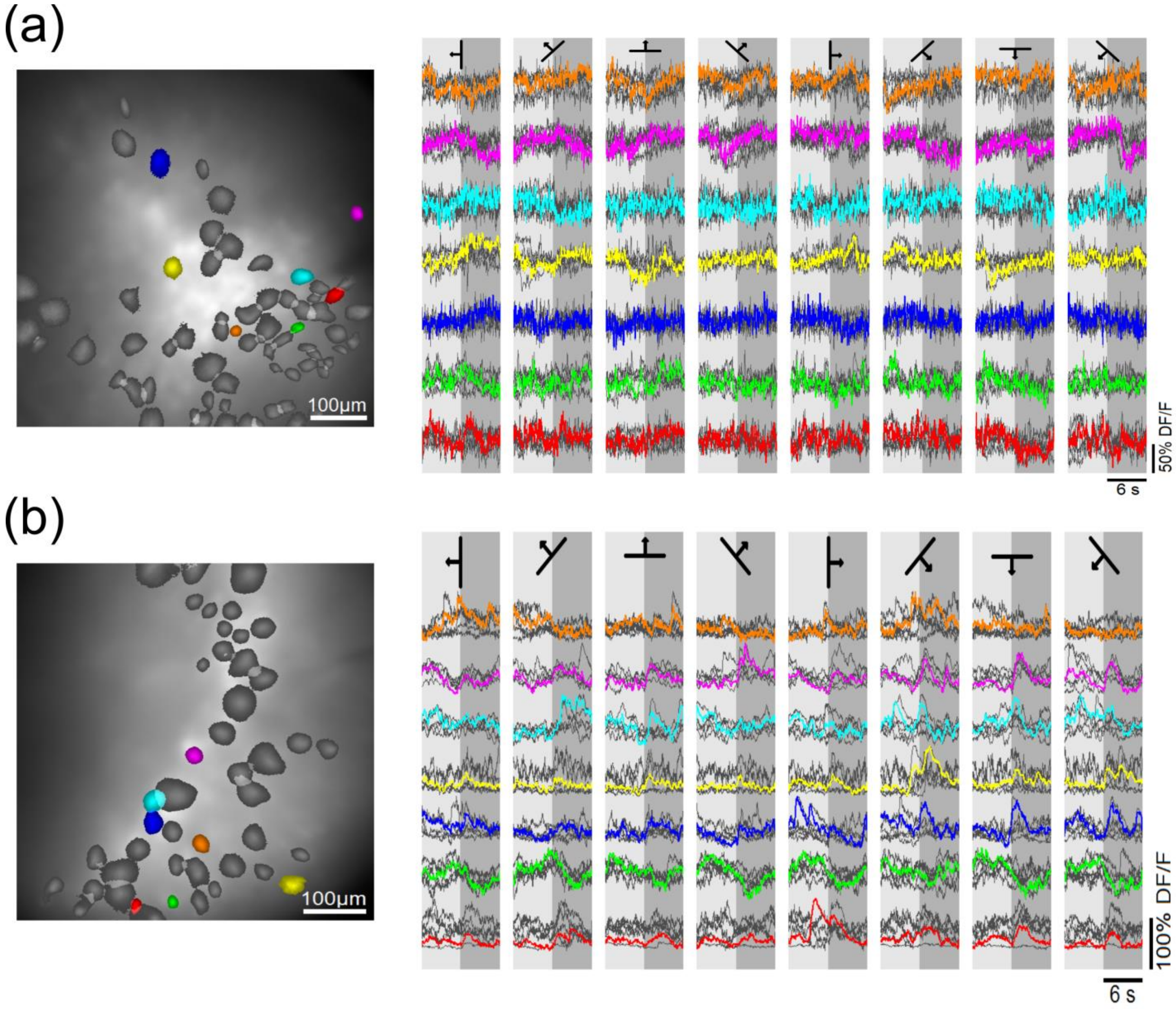

Supplementary Figure 18. Reaction of NTnC and GCaMP6s calcium indicators on complex visual stimuli *in vivo* registered with nVista HD. (a) Spatial filters and sample traces obtained from imaging session with light anesthetized mouse expressing NTnC; visual stimuli consisted of sinusoidal grating moving in 8 different directions (dark gray), separated by uniform blanc periods (light gray). Total 5 presentations were made for each direction (black traces); mean traces for each direction (colored traces) are overlaid. **(b)** Spatial filters and sample traces obtained from imaging session with mouse expressing GCaMP6s under the same conditions.

Supplementary Table 1. Characteristics of calcium responses for neurons expressing NTnC and GCaMP6s sensors during spontaneous activity of neuronal dissociated cultures.

Sensor	$\Delta F/F_0$	Rise half-time, msec	Decay half-time, msec
**NTnC**	0.20±0.02	500±200	1800±700
**GCaMP6s**	0.6±0.2	600±200	2500±1700

Supplementary Table 2. Electrophysiological characteristics for spontaneous activity of neuronal cultures plated on MEAs.

Culture	DIV	Median time intervals for individual distributions, sec	Median time intervals for summarized distribution, sec	Median number of spikes in burst for individual distributions	Median number of spikes in burst for summarized distribution
**non-transduced**	9	6.5; 7.1; 4.1;	4.8	183; 146; 228	187
	13	2.8; 4.0; 2.5	2.9	558; 446; 1078	613
	16	3.0; 1.8; 7.2	3.4	1024; 874; 1328	995
**GCaMP6s transduced**	9	5.2; 15.9; 9.5; 7.1	7.1	197; 129; 239; 128	164
	13	2.7; 2.7; 2.2; 2.8	2.5	738; 323; 352; 394	413
	16	2.2; 3.5; 2.8; 3.1	2.7	417; 363; 412; 273	375
**NTnC transduced**	9	9.1; 10.0; 8.5; 6.9	8.3	72; 102; 138; 124	103
	13	1.9; 1.5; 2.4 2.5	1.9	278; 383; 274; 300	321
	16	3.6; 4.0; 3.0; 2.4	3.1	416; 239; 477; 399	365

Supplementary Table 3. Characteristics of calcium responses in neurons expressing NTnC and GCaMP6s sensors in brain cortex of freely moving mice registered with nVista HD.

Properties		Proteins	
		**NTnC**	**GCaMP6s**
**Number of cells**	**assigned**	79	199
	**active** [a]	57	188
**Average activity frequency, spikes/s**		0.018	0.025
**Rise half-time, ms** [b]		300	250
**Decay half-time, s** [c]		2.5	1.7
**Peak $\Delta F/F_0$** [d]		0.012±0.004	0.03±0.02
**SNR** [e]		4.4±1.1	4.7±1.8

[a] cell was considered as active if its spiking threshold was >4MAD [b] rise half-time was calculated as a time interval between 4 MAD threshold crossing and peak of the mean spike. [c] decay half-time was determined as a time interval between the peak and half-peak on the back front of the mean spike. [d] peak $\Delta F/F_0$ was calculated as $(F-F_0)/F_0$, where F_0 is the baseline fluorescence signal averaged over the whole period of imaging. [d] Signal-to-noise ratio (SNR) was quantified as peak $\Delta F/F_0$ response over median absolute deviation (MAD), calculated for each cell over the whole period of imaging. [b,c,d,e] For calculation of these characteristics we considered 175 and 692 single spikes for NTnC and GCaMP6s, respectively. Mean values ± standard error of mean are given.

Supplementary Table 4. Characterization of *in vivo* calcium activities for neurons expressing NTnC and GCaMP6s sensors in brain cortex of anesthetized mice during presentation of simple visual stimuli registered with nVista HD.

**Properties**		**Proteins**	
		**NTnC**	**GCaMP6s**
**Number of cells**	**assigned**	64	54
	**active** [a]	10	50
**Spikes detected**		15	323
**Mean firing rate, spikes/s**		0.0037	0.02

[a] cell was considered as active if its spiking threshold was >4MAD. Visual stimuli consisted of 60W incandescent lamp and blue 465 nm LED pulses (Supplementary Figure 13)

Supplementary Table 5. Characterization of the *in vivo* calcium activities for neurons expressing NTnC and GCaMP6s sensors in brain cortex of anesthetized mice during presentation of complex visual stimuli registered with a NVista HD.

**Properties**		**Proteins**	
		**NTnC**	**GCaMP6s**
**Number of cells**	**assigned**	67	45
	**active** [a]	15	45
**Spikes detected**		34	374
**Mean firing rate, spikes/s**		0.004	0.014

[a] a cell was considered as active if its spiking threshold was >4MAD. Visual stimuli consisted of sinusoidal grating moving in 8 different directions, separated by uniform blanc periods (Supplementary Figure 13).

Supplementary Table 6. List of primers.

Primer	Primer sequence (5’-3’)
BglII-Neon forward	TCGAGATCTATGGTCTCAAAGGGAGAGGAGGACAACATGGCTAGTCTGCCTGCTACCCACGAACTGCATATTTTTGGCTC
1 reverse	TCATAACCGTCGTTTGGATTACCGGTGCCTTGGCCTACCATGTCGAAATCGACGCCATTTATGGAGCCAAAAATATGCAG
2 forward	CCAAACGACGGTTATGAGGAGCTGAACCTGAAGTCCACCAAGGGCGATCTGCAGTTCAGTCCCTGGATCCTGGTGCCG
3 reverse	CAGCCTGAAATGGTGACATCCCGTCTGGATAGGGAAGATACTGATGAAACCCGTACCCAATGTGCGGCACCAGGATCCAG
4 forward	GTCACCATTTCAGGCTGCAATGGTCGACGGGTCCGGATATCAGGTGCACCGCACTATGCAGTTTGAGGACGGTGCTTCAC
5 reverse	CCCTTCACCTGAGCTTCCCCTTTGATATGTGATCCTTCGTAGGTGTATCTGTAATTCACGGTCAGTGAAGCACCGTCCTC
6 forward	GAAGCTCAGGTGAAGGGCACTGGGTTCCCAGCTGACGGCCCTGTGATGACAAATTCCCTGACAGCCGCTGACTGGTGTAG
7 reverse	GGTGGTATAAGACCACTTAAATGTGGAAATGATAGTCTTATCATTGGGGTAAGTCTTCTTGGATCTACACCAGTCAGCGG
8 forward	GTGGTCTTATACCACCGGCAACGGCAAACGCTACCGGTCTACCGCTCGCACCACATACACATTCGCCAAACCAATGGCCG
9 reverse	CTTAGAATGTTTCAGCTCTGTTTTCCGAAAGACATACATCGGCTGATTTTTGAGGTAATTTGCGGCCATTGGTTTGGCG
10 forward	GCTGAAACATTCTAAGACCGAACTGAATTTTAAAGAGTGGCAAAAGGCCTTTACGGACGTGATGGGCATG
EcoRI-Neon reverse	TCGGAATTCCTTATACAGCTCGTCCATGCCCATCACGTCC
tTnC-BglII forward	CGAGATCTAGCGAAGAAGAGCTATCAGAGTGCTTTCGCATTTTTGACAAGGATGGAAATGGTTTCATCG
1 reverse	CTGTGAGCTGTTCTCCAGTAAGGCGGATGATGTCTCCAAATTCCTCCCTGTCGATGAAACCATTTCCATC
2 forward	CTGGAGAACAGCTCACAGATGAGGATCCCGATGAGATATTTGGAGATTCAGACACAGACAAAAATGGAAG
tTnC-EcoRI reverse	GCGAATTCCTACTGGACATTCTCCACCATCTTCAGGAACTCATCAAAGTCAATCCTTCCATTTTTGTCTGTG
NNeon-TnC forward	GACAAATTCCCTGACAGCCNNSNNSNNSAGCGAAGAAGAGCTATCAG
TnC-NNeon reverse	CTGATAGCTCTTCTTCGCTSNNSNNSNNGGCTGTCAGGGAATTTGTC
TnC-CNeon forward	GATGGTGGAGAATGTCCAGNNSNNSNNSGCTGACTGGTGTAGATCC
CNeon-TnC reverse	GGATCTACACCAGTCAGCSNNSNNSNNCTGGACATTCTCCACCATC
Neon-BglII-2 forward	CGAGATCTATGGTCTCAAAGGGAGAGGAG
Neon-EcoRI-2 reverse	GCGAATTCCTACTTATACAGCTCGTCCATGC
Neon-dTnC *forward*	GCAAATTCCCTGACAGCCNNSCCGAGCGAAGAAGAGCTATC
Neon-dTnC2 *forward*	GCAAATTCCCTGACAGCCNNSNNSAGCGAAGAAGAGCTATC
Neon-dTnC *reverse*	GATAGCTCTTCTTCGCTCGGSNNGGCTGTCAGGGAATTTGC
dTnC-Neon *forward*	GATGGTGGAGAATGTCCAGNNSNNSGCTGACTGGTGTAGATCC
dTnC-Neon *reverse*	GGATCTACACCAGTCAGCSNNSNNCTGGACATTCTCCACCATC
TnC-KpnI-NheI forward	CCGGTACCGGCTAGCGCCACCATGGTCTCAAAGGGAGAG
TnC-EcoRI-HindIII reverse	GTCAAGCTTGAATTCTTACTTATACAGCTCGTCCATG
Neon-dTnC forward	GCAAATTCCCTGACAGCCNNSCCGAGCGAAGAAGAGCTATC
Neon-dTnC2 forward	GCAAATTCCCTGACAGCCNNSNNSAGCGAAGAAGAGCTATC

Neon-dTnC reverse	GATAGCTCTTCTTCGCTCGGSNNGGCTGTCAGGGAATTTGC
dTnC-Neon forward	GATGGTGGAGAATGTCCAGNNSNNSGCTGACTGGTGTAGATCC
dTnC-Neon reverse	GGATCTACACCAGTCAGCSNNSNNCTGGACATTCTCCACCATC
TnC-166D+ forward	CTTTTGACAAGGATGGAGATGASGGTTTCATCGACAGGGAG
TnC-166D+ reverse	CTCCCTGTCGATGAAACCSTCATCTCCATCCTTGTCAAAAG
TnC-202D+ forward	GATTCAGACACAGACAAAAATGASGGAAGGATTGACTTTGATG
TnC-202D+ reverse	CATCAAAGTCAATCCTTCCSTCATTTTTGTCTGTGTCTGAATC

Supplementary Methods.

Libraries screening.

Screening of bacterial libraries was sequentially performed on Petri dishes, bacterial suspensions in a 96-well plate format, and purified proteins.

Primary screening of approximately 20,000 – 40,000 colonies of bacterial library expressing calcium sensors variants was performed on Petri dishes under fluorescent stereomicroscope Leica M205FA (Leica, Germany). Expression of the sensors in the colonies on Petri dishes was induced with 0.0002% arabinose for 16 h at 37°C and 24 h at room temperature (r.t.). Reaction of the sensors with calcium ions was further monitored under the fluorescent stereomicroscope Leica. Green and red fluorescence was registered by 480/40BP and 540/40BP excitation filters, respectively, and 535/40BP and 620/40BP emission filters, respectively. Fluorescence images of Petri dishes with bacterial colonies were snapped before and after spraying the plates with 100 mM EDTA, 100 mM Na_2HPO_4 at pH 7.4. Images obtained were analyzed using the ImageJ software and colonies having the highest brightness and contrast were picked up for further analysis.

Next, approximately 60-96 mutants selected through colonies analysis were analyzed on bacterial suspensions using 96-well Modulus™ II Microplate Reader (Turner Biosystems, USA). For this purpose, the best clones picked up from Petri dishes were grown in 200 uL aliquots of LB medium containing and 100 μg/ml ampicillin, 0.0002% arabinose, and 100 μM $CaCl_2$ for 12-16 h at 220 rpm and 37^0C and for 24 h at r.t. Bacterial suspensions containing 180 ml of 100 mM NaOAc pH 7.4 buffer supplemented with 100 mM $CaCl_2$ and 20 μl of bacterial culture were aliquoted onto 96-well plates. These suspensions were incubated at r.t. for 1 h with measurement of the fluorescence signal. Afterwards, EDTA solution was added until a final concentration of 0.4mM, followed by fluorescence registration for 10 min. Next, a solution of $CaCl_2$ was added until a final concentration of 5mM, followed by fluorescence recording for 10 min. Data collected was analyzed using the Origin 6.0 software, as plots of dependence of fluorescence vs time. Clones having the highest brightness and contrast in response to addition of $CaCl_2$ and EDTA were selected.

The best clones found on bacterial suspensions were subsequently grown for protein purification in LB supplemented with 0.0002% arabinose, 100 μg/ml ampicillin and 100 μM $CaCl_2$ overnight at 37^0C, 220 rpm. The cultures were centrifuged at 1,640 g

for 15 min. The cell pellets were resuspended in BiPer solution (Thermo Scientific, USA) containing 1 mg/mL lysosyme, and 20 u/mL DNAse I (Invitrogen, USA). The recombinant proteins were purified using Ni-NTA resin (Qiagen, USA). Purified proteins were characterized for brightness (product of quantum yield and extinction coefficient) and contrast as described below. Clones exhibited increased brightness and contrast compared to clones from previous round of mutagenesis were subjected to the next round of random mutagenesis.

Protein characterization.

For determination of the pH dependence, NTnC and GCaMP6s proteins were dialyzed in Ca^{2+}-free buffer (10 mM Tris-HCl, 100 mM KCl, 10 mM EDTA, pH 7.2) or in Ca^{2+} containing buffer (10 mM Tris-HCl, 100 mM KCl, 10 mM $CaCl_2$, pH 7.2). Next, they were diluted 1:100 into a series of pH adjusted buffers (30 mM citric acid, 30 mM borax, or 30 mM NaCl) with pH values ranging from 9 to 3 in 0.5 pH units interval in a 96-well black clear bottom plate (Thermo Scientific, USA), as described in the original paper[1]. Fluorescence was measured using a Modulus™ II Microplate Reader (TurnerBiosystems, USA).

Photobleaching experiments were performed with suspensions of purified proteins in mineral oil, as previously described[2]. Briefly, the kinetics of photobleaching was measured using purified proteins dialyzed in Ca^{2+}-free (10 mM Tris-HCl, 100 mM KCl, 10 mM EDTA, pH 7.2) or in Ca^{2+} containing buffers (10 mM Tris-HCl, 100 mM KCl, 10 mM Ca-EDTA, pH 7.2) at a 1 mg/ml concentration, in aqueous microdroplets in mineral oil using Zeiss Axio Imager Z2 microscope (Zeiss, Germany) equipped with a 120 W mercury short-arc lamp (LEJ, Germany), a 63x 1.4 NA oil immersion objective lens (PlanApo, Zeiss, Germany), a 470/40BP excitation filter, a FT 495 beam splitter, and 525/50BP emission filters. Light power density was measured at a rear focal plane of the objective lens. The times to photobleach from 1000 down to 500 emitted photons per second were calculated according to standard procedures[3]. In brief, the averaged raw data were corrected for a spectral output of the lamp, transmission profiles of the excitation filter and dichroic mirror, and absorbance spectra of the respective green fluorescent proteins and their quantum yields. EGFP protein that has been characterized according to this procedure in [3] was used as a reference.

To study protein maturation, BW25113 bacteria transformed with the pBAD/HisB-TorA-NTnC or pBAD/HisB-mEGFP plasmids were grown in LB medium supplemented with ampicillin at 37°C overnight. The next morning 0.2% arabinose was added to bacterial cells. Upon induction of protein expression, bacterial cultures were grown at

37°C in 50 ml tubes filled to the brim and tightly sealed to restrict oxygen supply. After 2 hours, the cultures were centrifuged in the same tightly closed tubes. After opening the tubes, the bacteria were sonicated in PBS buffer and the resulting proteins were purified using Ni-NTA resin within 10 min, with all procedures and buffers at or below 4°C. Protein maturation occurred in Ca^{2+}-free or Ca^{2+}-saturated buffers at 37°C. Green fluorescence signal of the proteins was monitored using a CM2203 spectrofluorometer (Solar, Belarus).

Mammalian Plasmid Construction.

In order to construct the pAAV-*CAG*-TagRFP plasmid, the TagRFP (Evrogen) gene was PCR amplified as an NheI-EcoRI fragment and swapped with the iRFP-P2A-EGFP gene in the pAAV-*CAG*-iRFP-P2A-mCherry vector. In order to construct pAAV-*CAG*-NTnC, the pAAV-*CAG*-GCaMP6s and pAAV-*CAG*-R-GECO1 plasmids TagRFP, NTnC, GCaMP6s and R-GECO1 were PCR amplified as NheI-EcoRI and BamHI-EcoRI fragments and swapped with the iRFP-P2A-EGFP gene in the pAAV-*CAG*-iRFP-P2A-mCherry vector.

rAAV particles production and isolation.

The rAAV particles were purified as described in original paper[4], with some modifications. Briefly, HEK293T cells were grown in six 15 cm diameter dishes (Greiner Bio-One, Austria) filled with 25 ml of standard DMEM medium supplemented with 10% FBS, GlutaMaxI, 50 U/ml penicillin, and 50 U/ml streptomycin and incubated at 37°C to cell density of 60-80%. Cells were then transfected using the calcium phosphate method with pAAV-DJ (168 μg) and pHelper (168 μg) plasmids mixed with either 168 μg of pAAV-*CAG*-NTnC, pAAV-*CAG*-GCaMP6s, pAAV-*CAG*-RGECO, or pAAV-*CAG*-NTnC/166D+/202D+ plasmids. For transfection, we sequentially mixed 22 ml of aqueous plasmid solution in 250 mM $CaCl_2$ with 2xHBS buffer and incubated for 20 min at RT. The solution was then added drop-wise to the cell cultures in DMEM/GlutaMaxI/p/s without FBS. Cells were incubated with the transfection mix for 16-20 h at 5% CO_2 and 37°C. After the medium was exchanged for DMEM/10% FBS/GlutaMaxI/p/s and cells were cultured for an additional 48 hours. Afterwards, the cells were washed with 60 ml of DPBS, treated with 24 ml of 0.25% trypsine-EDTA for 5 min at 37 degrees, resuspended in 10ml of DMEM medium, transferred into 50-mL tubes, and centrifuged at 1,640 g for 1 min. Pellets were then resuspended in 60 ml 100 mM NaCl, 20 mM Tris-HCl, pH8.0. Next, we added 10% sodium deoxycholate until a final concentration of 0.5% and benzonase until a final concentration of 25 u/ml. After this, cells were incubated for 1 hour at 220 rpm and 37°C. Cellular debris was removed

by centrifugation at 1,640 g for 15 min. Viral particles were bound with 0.5 ml of HiTrap Heparin (GE Healthcare, UK) in 50 mL tubes in batch experiments for 30-60 min, at r.t. with mixing. After the resin was pelleted down by centrifugation at 1,640 g for 2 min and washed with 12 ml of 100 mM NaCl, 20 mM Tris-HCl at pH 8.0. The resin was replaced into the column and particles were eluted in 2.5 mL of 500 mM NaCl, 20mM Tris-HCl, pH8.0. Collected particles then were concentrated on an Optima^{TM} MAX-XP ultracentrifuge (Beckman Coulter, USA) for 20-24 h at 80,000 g and 15-16°C until a final volume of ~100 μl.

For titration of viral particles HEK293T cells were seeded onto MatTek glass bottom dishes and grown till 60-70% confluency. Each dish was infected with a serial dilution of AAV vector; two dishes were used for each dilution. After 48-72 h, cellular cultures were imaged using an Andor XDi Technology Revolution multi-point confocal system (Andor, UK). The number of transduced cells in the dishes with the highest dilution factor, but still containing infected cells, were counted and titer was determined as the average number of transduced cells multiplied by the dilution factor.

Culture and electrophysiology of neuronal cultures plated on MEAs.

Plating and culture of dissociated cortical neurons isolated from new born C57BL/6 pups (P0) was performed in 60 channel MEAs (MCS, Germany), according to the following procedure. MEAs were sterilized by their incubation in 70% ethanol for 1 hour followed by UV-illumination in a laminar flow hood for 5 hours and then treated with 0.05% polyethylenimine that provides adhesion of neurons to the surface. We further decapitated the new born pups with scissors. In sterile conditions (under laminar flow) the brains tissue was extracted and immediately placed in chilled dissection buffer. Under a microscope we dissect the cortex from the two hemispheres sequentially, using forceps and a scalpel. Extracted cortexes were separated from blood vessels and placed into the new dish with chilled dissection buffer. After isolation of the cortexes from all mice, we transferred all of them by forceps into a new dish and chopped in a drop of buffer by scalpel. Chopped tissue was transferred into a tube with 2 ml 0.25% trypsin (Invitrogen 25200-056), pre-warmed to 37°C, and placed in incubator for 20 min at 37°C. Pre-warmed to 37°C complete culture medium (Neurobasal medium (Invitrogen, USA) supplemented with bioactive B27 supplement (Invitrogen, USA) and glutamine (Invitrogen, USA) was added into the tube with the trypsinized tissue, followed by precipitation of the cells by centrifugation at 430 g for 5 min. Supernatant was aspirated and cells were resuspended in 9ml of complete culture medium followed by centrifugation at 430 g for 5 min. The supernatant was removed and cells were

resuspended in 1ml of complete culture medium. After we counted the cells and adjusted their concentration to 3 million/ml. 50 μl of cells were plated on a matrix or glasses pre-treated with polyethylenimine. The density of the cell culture was at least 1200 cells/mm. MEAs were placed in CO_2 incubator for 30 min, 37°C. After incubation we added 1 ml of complete culture medium pre-warmed to 37°C dropwise and cells were placed in a CO_2-incubator. Cell culture was conducted in a CO_2-incubator at 37°C and 5% CO_2. Complete culture medium was exchanged for fresh one (one half of the volume was replaced for equal volume of fresh complete medium) in 24 h after cells plating onto the glass and once 3 days afterwards.

Furthermore, we transduced and characterized the electrical properties of the cultures. Transduction of neuronal cultures was performed on the 6th day after culture plating on matrixes by addition of 1-2 μl of solution with viral particles carrying genes for fluorescent sensors. Registration of electrical activity for neuronal cultures was accomplished by a multielectrode registration system *in vitro* MEA60 System (MCS GmbH, Germany). This system included MEA1060-Up-BC-Standard set (composed of MEA1060-Up-BC preamplifier, FA60S-BC amplifier, C68x1M cable); USB-ME256-System for digitizing and data entry; STG4008 stumuli generator; TC02 controller of thermostates; PS40W power source; MPU30 adapter; set of cables for synchronization, stimulation and ground connection; adapter for membranes; membranes; MEA with 60 electrodes of 30 μm diameter and 200 μm inter-electrode distance (200/30-ITO-gr or 200/30-ITO-stim-gr).

Recorded signals from neuronal cultures were filtered and spikes were detected if the signal amplitude exceeded 4 standard deviations of all signal from one day. A burst event was defined as a train of high frequency spiking with a duration of 0.1-3 s recorded on a single electrode. The burst is usually accompanied by a low frequency 1-5Hz component. The detection of bursts was carried out by identifying of the low frequency component in a pre-determined window and detecting spikes around this component. Durations between bursts were found by calculation of time interval from the end of one burst to the start of the next one.

Analysis of V1 two-photon functional imaging

Image analysis was performed in FIJI[5] (www.fiji.sc). Regions of interest (ROIs) corresponding to identifiable cell bodies were selected manually. XY motion artifacts were corrected with TurboReg plugins[6]. The fluorescence time course was measured by averaging all pixels within the ROI with the Time series analyzer plugin. The $\Delta F/F_0$ of

each trial was calculated as (F-F0)/F_0, where F_0 was averaged over a 10 s period for before the start of stimulus.

Image analysis with a NVista HD miniature microscope

All processing of calcium imaging data was made using the Mosaic software (Inscopix Inc., USA), and custom MATLAB scripts. First, all movies were spatially down-sampled by a factor of 2 in order to increase computation speed. Then, rigid body registration was made using a Mosaic routine based on TurboReg algorithm[6] to correct lateral displacements of the focal plane. After this, $\Delta F/F$ normalization was applied to the movies: $\Delta F/F = (F - F_0)/F_0$, where F_0 is intensity value for each pixel, averaged over time. For cell identification, spatial filters corresponding to individual cells were obtained using principal component analysis, followed by independent component analysis (PCA/ICA [7]). After a threshold of 50% of the filter's maximum intensity was applied to each filter, and all pixels with values lower than a threshold of one were set to zero. Filters with thresholds with low circularity, noisy appearance, and non-smooth borders were manually excluded from further analysis. After this, activity traces were extracted by applying threshold filters to $\Delta F/F$ movies. To correct neuropil contamination, for each trace the neuropil correction was made according to following equation: $F_{true}(t) = F(t) - F_{neuropil}(t)$, where F(t) is an extracted trace, and $F_{neuropil}(t)$ is a mean trace of all pixels inside 25 µm circle, with center at the brightest point of threshold filter; all threshold filters were excluded from this circle. Also, in movies captured in experiments with light and grating demonstrations, strong baseline fluctuations were observed; to correct them, the median trace (20 s sliding window) was subtracted from each obtained trace.

Calcium events (spikes) were detected whenever difference between a trace amplitude and it median value crossed the threshold of ±4 median absolute deviations (MADs, were calculated for each cell over the whole trace; + for GCaMP6s, - for NTnC). To avoid false detection, an event was taken in account only if all values during the previous 0.5s period did not exceeded threshold. In order to exclude peaks, faster than typical calcium events, candidate spikes were considered only if all values during the 1s period after threshold crossing exceeded the threshold. To prevent detection of multiple peaks within the same event, no spikes could be detected closer than 1s to each other. In order to calculate mean spikes and asses their parameters (Fig. 6b), only single events were taken; spike were considered only if there were no any other spikes in a 4 s interval before and 10 s interval after it. Rise half-time was measured as the time from 4 MAD threshold crossings to the peak of the mean spike. Decay half-time was calculated

as the time from the peak to half-peak on the back front of the mean spike. Finally, signal-to-noise ratio (SNR) was quantified as peak $\Delta F/F$ response over 1 MAD.

Animal care. All methods for animal care and use were approved by the National Research Center "Kurchatov Institute" Committee on Animal Care (protocol No 1, 07th September 2015) and were in accordance with the *Russian* Federation Order Requirements N *267* M3 and the National Institutes of Health Guide for the Care and Use of Laboratory Animals. 29 C57BL/6 mice were used in this study, ages P0 and ~2-6 months old. Mice were used without regard for gender.

Supplementary Results.

To test NTnC sensors for *in vivo* registration of neuronal activity with aNVista head-mounting microscope we visualized neuronal calcium activity in the visual cortex brain region of anesthetized awake mice in response to simple and complex visual stimuli. We installed a NVista miniaturized microscope under the layer 2/3 of the visual brain cortex of mice transduced with rAAV particles carrying NTnC and control GCaMP6s green calcium indicators under control of the CAG promoter (Fig. 6a).

First, we presented light pulses from an incandescent lamp and blue LED array as simple visual stimuli to slightly anesthetized awake mice. For both NTnC and GCaMP6s mice we could not detect reproducible neuronal activity to either white or blue light pulses (Supplementary Fig. 13 and Supplementary Table 4). In the case of the GCaMP6s mouse, we detected neuronal activities that sometimes coincided with the beginning and end of light pulses. For the NTnC mouse, we registered significantly less calcium neuronal activity that also only sometimes coincided with white light and did not see reaction to blue light.

Next, we attempted to visualize the neuronal calcium activity in response to grating moving in 8 different directions as complex visual stimuli to slightly anesthetized awake mice. For both NTnC and GCaMP6s mice we could not detect neuronal responses that were orientation specific and reproducible across five trials (Supplementary Fig. 14 and Supplementary Table 5). Failure to find orientation specific calcium neuronal activity was probably connected with anesthesia and/or the limited field of view of the NVista microscope. Indeed, with non-anesthetized mice and other type of *in vivo* microscopy, we successfully found neurons responding to specific orientation of grating (Fig. 5).

Hence, in an *in vivo* model with slightly anesthetized mice and NVista miniaturized microscope we observed less calcium neuronal activity, compared with experiments using non-anesthetized mice (Main text) and, consequently, in order to access calcium neuronal dynamics *in vivo* it is necessary to work with non-anesthetized animals.

Supplementary References

1. Zhao, Y. et al. An expanded palette of genetically encoded Ca(2) indicators. *Science* **333**, 1888-1891 (2011).
2. Subach, O.M. et al. Conversion of red fluorescent protein into a bright blue probe. *Chem. Biol.* **15**, 1116-1124 (2008).
3. Shaner, N.C., Steinbach, P.A. & Tsien, R.Y. A guide to choosing fluorescent proteins. *Nat Methods* **2**, 905-909 (2005).
4. McClure, C., Cole, K.L., Wulff, P., Klugmann, M. & Murray, A.J. Production and titering of recombinant adeno-associated viral vectors. *J Vis Exp*, e3348 (2011).
5. Schindelin, J. et al. Fiji: an open-source platform for biological-image analysis. *Nature methods* **9**, 676-682 (2012).
6. Thevenaz, P., Ruttimann, U.E. & Unser, M. A pyramid approach to subpixel registration based on intensity. *IEEE Trans Image Process* **7**, 27-41 (1998).
7. Mukamel, E.A., Nimmerjahn, A. & Schnitzer, M.J. Automated analysis of cellular signals from large-scale calcium imaging data. *Neuron* **63**, 747-760 (2009).
